# Transcription-replication conflict resolution by nuclear RNA interference

**DOI:** 10.1016/j.molcel.2025.10.003

**Published:** 2025-10-28

**Authors:** Teri Cheng, Benjamin Roche, Farida Abderahmane, Leila Touat-Todeschini, Karine Fréon, Asad A. Lakhani, Sonali Bhattacharjee, Liam G. Spielmann, Emerson Jenen, Christine Choi, Jie Ren, André Verdel, Sarah A.E. Lambert, Robert A. Martienssen

**Affiliations:** 1 Howard Hughes Medical Institute, Cold Spring Harbor Laboratory, 1 Bungtown Road, Cold Spring Harbor, NY 11724, USA; 2 School of Biological Sciences, Cold Spring Harbor Laboratory, 1 Bungtown Road, Cold Spring Harbor, NY 11724, USA; 3 Team RNA, Epigenetics and Stress, Institut for Advanced Biosciences, Université Grenoble Alpes, Inserm U1209, CNRS UMR 5309, Grenoble, France; 4 Institut Curie, Université PSL, CNRS UMR3348, 91400 Orsay, France; 5 Université Paris-Saclay, CNRS UMR3348, 91400 Orsay, France; 6 Equipe Labélisée Ligue Nationale Contre le cancer, 91400 Orsay, France; 7 Present address: Department of Biomedical Sciences, School of Medicine & Health Science, University of North Dakota, Grand Forks, ND 58202, USA; 8 Present address: State Key Laboratory of RNA Innovation, Science and Engineering, CAS Center for Excellence in Molecular Cell Science, Shanghai Institute of Biochemistry and Cell Biology, University of Chinese Academy of Sciences, Chinese Academy of Sciences, Shanghai, 200031, China; 9 Lead contact

## Abstract

Nuclear RNA interference (RNAi) is required for heterochromatin silencing, but Dicer also promotes genome stability by releasing RNA polymerase at sites of replication stress. R-loops are three-stranded DNA:RNA structures that accumulate at transcription-replication (T-R) collisions. We show that in RNase H-deficient cells, which accumulate pathological R-loops, Dcr1 processes R-loops at transcriptional start sites (TSSs) and end sites (TESs), releasing paused RNA polymerase and accounting for small RNAs (sRNAs) resembling DNA-damage-associated sense sRNAs (sdRNAs) found in cancer cells. Genetic evidence implicates nascent transcription-associated R-loops in genome instability in the absence of Dicer, with the helicase domain providing catalytic function reminiscent of related archaeal helicases involved in replication. The RNase H homolog Argonaute (Ago1) promotes genome instability by binding R-loops, and its removal relieves replication stress. Analysis of replication intermediates, DNA and RNA 3^’^ ends, and fork processivity genome wide indicates Dicer resolves head-on T-R collisions, consistent with an ancient origin in DNA replication.

## In brief

Nuclear RNA interference limits genome instability beyond centromere function, though the mechanism was largely unknown. In this study, Cheng et al. discovered that, in *S. pombe*, Dicer (Dcr1) terminates RNA polymerase II at promoter-proximal pause sites when R-loops accumulate, which would otherwise cause head-on collision with the replication machinery.

## Graphical Abstract

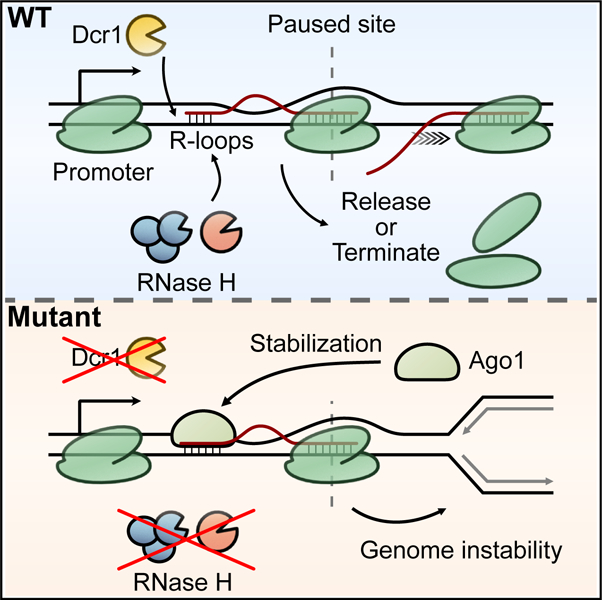

## INTRODUCTION

RNA interference (RNAi), widely known for its post-transcriptional gene regulatory functions, has an ancient and fundamental role in heterochromatic silencing and genome stability.^1–5^ In the fission yeast *Schizosaccharomyces pombe*, disruption of any of the RNAi components–Dicer (Dcr1), Argonaute (Ago1), or the RNA-dependent RNA polymerase (Rdp1)–impairs pericentromeric heterochromatin formation and cohesin recruitment, leading to chromosome mis-segregation.^6,7^ Beyond centromere functions, RNAi also assists in DNA damage response (DDR),^8^ and in plants and animals, small RNAs (sRNAs) are generated proximal to damage sites that are thought to promote repair.^9,10^

We previously discovered that Dcr1 releases RNA polymerase II (RNA Pol II) to limit transcription-replication (T-R) stress.^3,4,11^ This function appeared to be independent of canonical RNAi. For example, only *dcr1*Δ, but not *ago1*Δ, accumulated Rad52 foci, indicating persistent DNA damage,^3^ and *dcr1*Δ progressively lost ribosomal DNA (rDNA) copies over meiotic generations, but this was not observed in the RNase III catalytic dead allele *dcr1–5*.^4^ Similar Dicer-specific, sRNA-independent chromosomal phenotypes were also documented in mouse embryonic stem cells (mESCs),^12^ suggesting broad conservation. Nevertheless, how transcription is regulated by Dicer, and how this prevents genome instability, remains poorly understood.

R-loops are three-stranded nucleic acid structures consisting of a DNA:RNA (D:R) hybrid with the displaced single-stranded DNA (ssDNA) and are thought to occur predominantly co-transcriptionally.^13^ Physiological R-loops can be beneficial, but misregulated R-loops can become pathological, obstructing DNA replication and causing DNA damage and genome instability.^14^ R-loops are therefore extensively regulated, with two conserved ribonucleases, RNase H1 and RNase H2, dedicated to degrading the RNA moiety of D:R hybrids.^15^ They operate partly redundantly,^16^ and in the double-mutant *rnh1*Δ *rnh201*Δ, cells become hypersensitive to genotoxic agents such as hydroxyurea (HU) and camptothecin (CPT),^17^ underscoring the importance of R-loop removal to genome stability. Unresolved R-loops obstruct replisome progression, slowing or completely arresting replication forks, requiring rescue from opposite forks or restart via the recombination-dependent replication (RDR) mechanism, which is error prone.^18^ Here, we set out to determine the mechanism of Dcr1 in limiting T-R stress, especially in contexts where R-loops accumulate, as well as the consequence to genome maintenance in the absence of Dcr1.

## RESULTS

### R-loop-induced T-R stress in the absence of Dcr1

**Figure 1.**
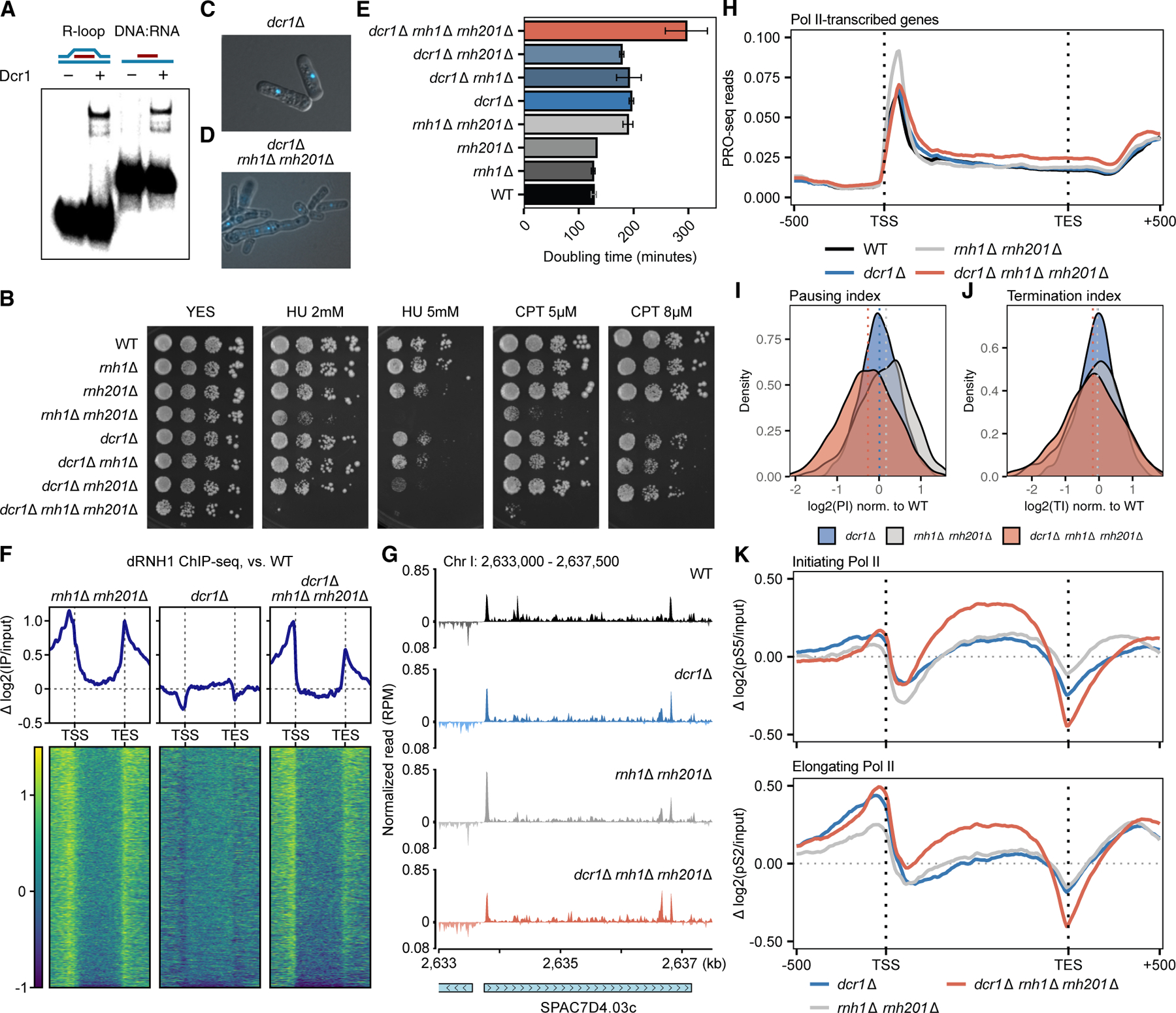
Dicer prevents unscheduled release of paused RNA Pol II in the presence of R-loops, which results in replication stress (A) Electrophoretic mobility shift assay (EMSA) of Dcr1 binding to R-loops and D:R hybrids *in vitro*. (B) Spot growth assays of WT and combinations of *rnh1*Δ, *rnh201*Δ, and *dcr1*Δ using 10-fold dilutions on YES plates without supplement or with indicated doses of the genotoxic agents, hydroxyurea (HU) and camptothecin (CPT). (C and D) Representative images of *dcr1*Δ (C) and *dcr1*Δ *rnh1*Δ *rnh201*Δ triple mutant (D) cells with DAPI staining of DNA. (E) Doubling time of WT and combinations of *rnh1*Δ, *rnh201*Δ, and *dcr1*Δ. *n* = 2; error bars represent standard deviation. (F) Heatmaps of R-loop levels of *rnh1*Δ *rnh201*Δ, *dcr1*Δ, and *dcr1*Δ *rnh1*Δ *rnh201*Δ relative to WT. dRNH1 ChIP-seq reads of individual genotypes were first normalized per million reads (RPM), followed by log_2_ normalization to input. The track was then subtracted with WT. The heatmap shows all RNA Pol II-transcribed genes (*n* = 4,346), collapsing gene bodies and with 500 bp upstream of annotated transcription start sites (TSSs) and 500 bp downstream of transcription end sites (TESs). (G) Representative genome track of normalized PRO-seq data of WT (black), *dcr1*Δ (blue), *rnh1*Δ *rnh201*Δ (gray), and triple mutant (red). Note the peak at the TSS of the gene SPAC7D4.03c and the pile-up toward the end of the transcript. (H) Detection of paused RNA polymerase by 3^’^-OH RNA ends in asynchronized WT (black), *dcr1*Δ (blue), *rnh1*Δ *rnh201*Δ (gray), and triple mutant (red) cells. PRO-seq reads were normalized per million reads (RPM) and mapped to genome-wide metaplots of collapsed gene bodies plus 500 bp upstream of annotated TSSs and 500 bp downstream of TESs for all RNA Pol II-transcribed genes (*n* = 4,346). (I) Density plot of transcriptional pausing indices (PIs) after normalization to WT, based on PRO-seq reads from (H). Dotted lines represent the median for each genotype. (J) Density plot of transcriptional termination indices (TIs) of well-isolated genes (see STAR Methods) after normalization to WT based on PRO-seq reads from (H). Dotted lines represent the median for each genotype. (K) Detection of initiating RNA Pol II (top) and elongating RNA Pol II (bottom) via phospho-serine-5 and phosphor-serine-2 ChIP-seq, respectively. Reads were mapped to all RNA Pol II-transcribed genes and normalized as in (H).

In *S. pombe*, R-loops accumulate in *dcr1*Δ around replication origins in pericentromeric repeats,^4^ where replication forks stall,^3^ suggesting a role for Dcr1 in T-R collision. We therefore purified *S. pombe* Dcr1 and performed binding assays and confirmed that *S. pombe* Dcr1 binds R-loops and D:R hybrids *in vitro* (Figure 1A), but not D-loops, and in an ATP-independent manner (Figures S1A and S1B). Human Dicer has also been shown to bind and process R-loops *in vitro*, and both R-loops and asymmetric forks can be detected in HeLa cells following Dicer knock-down.^19^ We quantified this binding using microscale thermophoresis, with a higher (∼10×) affinity of our *S. pombe* Dcr1 to R-loops than human Dicer obtained commercially (Figures S1C and S1D), though the affinity might vary due to differences in purity and activity. To investigate the role of Dicer in processing R-loops *in vivo*, we created higher-order mutants with RNase H1 and RNase H2, which accumulate R-loops in *S. pombe*.^20^ Strikingly, *dcr1*Δ induced HU- and CPT-hypersensitivity in these RNase H-deficient cells (Figure 1B). The negative interaction also manifested in elongated, multinucleate cells (Figures 1C and 1D), with a substantially longer doubling time (Figure 1E). As RNase H mutants have an extended S phase with elongated cells due to replication-dependent DNA damage,^17,21^ the results suggested that Dcr1 has a role in promoting faithful replication. We noted a stronger genetic interaction between *dcr1*Δ and *rnh201*Δ than with *rnh1*Δ (Figure 1B). This might be due to the additional roles of RNase H2 in double-stranded break (DSB) repair^16^ and replication restart.^22^

R-loops can be detected genome wide using the S9.6 antibody. However, this antibody is thought to cross-react with double-stranded RNA (dsRNA) and also fails to detect transient R-loops, such as those formed proximal to the promoter.^23^ Instead, we probed R-loops genome wide by inducing the expression of catalytically dead, tagged human RNase H1 followed by chromatin immunoprecipitation followed by sequencing (ChIP-seq),^20^ a technique that captures promoter proximal R-loops that are normally processed by RNase H. As expected, in RNase H-deficient cells, R-loops accumulated around transcription start and end sites (TSSs and TESs; Figure 1F). In *dcr1*Δ cells, however, R-loops were slightly depleted. Crucially, the R-loop level in the triple mutant appeared to be additive (Figure 1F), suggesting that the negative interaction between Dcr1 and RNase H could not simply be explained by Dcr1 being involved in directly processing R-loops.

Consistent with this idea, overexpressing *rnh1*^+^ alone did not rescue *dcr1*Δ (Figures S1E and S1F). We also tested interaction between *dcr1*Δ and orthologs of the human D:R helicase senataxin (SETX),^24^ *sen1*, and *dbl8*. We observed no obvious growth defects under the conditions tested for both *sen1*Δ and *dbl8*Δ, nor any substantial negative interaction between *dcr1*Δ and *dbl8*Δ (Figures S1G and S1H). Canonical RNAi mutants have defective centromere function and are sensitive to the microtubule-destabilizing drug thiabendazole (TBZ).^7^ We found neither RNase H, Sen1, nor Dbl8 influenced the TBZ sensitivity of *dcr1*Δ (Figures S1F–S1H), suggesting the phenotypes were independent of canonical RNAi. Unexpectedly, *dcr1*Δ *sen1*Δ double mutants were more resistant to CPT than *dcr1*Δ cells (Figure S1G). Considering the known transcription and replication roles of SETX,^24–26^ we noted that in *S. pombe* Sen1 predominantly interacts with RNA Pol III, and Dbl8 with RNA Pol I.^27^ Consistently, while Dcr1 plays a critical role in releasing RNA Pol I during cellular quiescence,^11^ it releases RNA Pol II from highly transcribed loci in cycling cells.^4^ As Dcr1 also terminates transcription at tDNA loci,^4^ the CPT resistance in *dcr1*Δ *sen1*Δ double mutant might reflect such additional function of Dcr1. Nevertheless, the ChIP-seq data and the differences in genetic interactions between *dcr1* and other R-loop processors suggested Dcr1 did not limit T-R stress by simply removing R-loops.

### Global R-loop-induced pausing defect is Dcr1-dependent

To investigate the mechanism by which RNA Pol II transcription is misregulated in *dcr1*Δ, we performed precision run-on sequencing (PRO-seq)^28^ on the mutant series to study nascent transcription at near single-nucleotide resolution. Immediately downstream of promoters, RNA Pol II is frequently held in a paused state by associating with the negative elongation factor (NELF) and the DRB sensitivity-inducing factor (DSIF) complexes.^29^ RNA Pol II is then either licensed into productive elongation through the action of the positive transcription elongation factor b (P-TEFb) complex or terminated via a recently described function of the Integrator complex, of which the INTS11 subunit mediates RNA endonucleolytic cleavage, releasing RNA Pol II,^30–32^ and promoter-proximal pausing is now known to be a critical checkpoint to regulate gene expression.^29^

*S. pombe* retains DSIF (Spt4/5) and P-TEFb (Cdk9), but lacks NELF and the Integrator complex. Nevertheless, global PRO-seq profiles revealed RNA Pol II is also paused in *S. pombe* (Figures 1G and 1H), as previously described.^33^ The extent of pausing and termination can be quantified with pausing and termination indices, respectively (PIs and TIs; see STAR Methods).^33^ Whereas the extent of termination was similar between the mutants, we observed a global pausing increase in *rnh1*Δ *rnh201*Δ (Figures 1H–1J, S1I, and S1J), suggesting efficient R-loop processing was required for pause release.^32,34^ Accordingly, promoter R-loops accumulated globally in *rnh1*Δ *rnh201*Δ (Figure 1F), indicating paused RNA Pol II pile-up as a signature of inefficient R-loop removal. The pausing defect was Dcr1-dependent, as further deleting *dcr1* in the double-mutant *rnh1*Δ *rnh201*Δ led to a decrease in PI of ∼80% of RNA Pol II-transcribed genes (Figure S1K). *dcr1*Δ alone had a wild-type (WT) level of pausing, and in the triple mutant, we observed a pause level below that of WT (Figures 1I and S1I). This was caused by higher read counts along the gene body (Figure 1H), indicating unscheduled pause escape. The pause defect was more drastic in the top 10% paused genes (Figure S1L) and was correlated with expression level (Figure S1M). We concluded that Dcr1 had a global function in limiting unscheduled RNA Pol II pause release, but especially in genes where promoter R-loops are actively resolved by RNase H. Supporting this idea, RNA Pol II carrying the serine-5 phosphorylation–which marks early-initiating polymerases^35^–was enriched across the gene body in the triple mutant (Figure 1K), which we proposed to be the consequence of pausing misregulation.

To study whether the pausing defect in *dcr1Δ* would affect gene expression, we profiled gene expression on asynchronous log-phase cells with total RNA sequencing (RNA-seq). In *dcr1*Δ single mutants, *rnh1*Δ *rnh201*Δ double mutants, and the triple mutant, genes were mostly up- rather than downregulated (Figure S2A), but as previously described,^36^ *dcr1* deletion did not drastically affect the transcriptome–only ∼2.7% of annotated transcripts were upregulated (Figure S2A, orange dots). Centromeric transcripts were reproducibly upregulated in both *dcr1*Δ and the triple mutant (Figure S2A, blue dots). By contrast, we observed no changes to genes involved in transcription elongation and termination (Figure S2A, yellow dots), arguing against the pause defect in our mutants being due to misregulation of such factors. *dcr1*Δ-induced genes were not enriched in any particular biological process, but this group of genes was also upregulated in *rnh1*Δ *rnh201*Δ and was even more deregulated in the triple mutant (Figures S2B and S2C). These genes were lowly expressed in WT. Nevertheless, the overlap suggests a shared regulatory mechanism that involved both Dcr1 and RNase H. We also looked for genes that were specifically misregulated in the triple mutant but not in *dcr1*Δ nor in *rnh1*Δ *rnh201*Δ cells (see STAR Methods). Despite a relaxed selection criterion, only 15 genes were found to be upregulated (Figure S2D). Of note, 6 genes (*tef101*, *tef102*, *tef103*, *SPAC29A4.02c*, *tef3*, and *cpc2*) were linked to translation elongation, suggesting that in the triple mutant, despite a relatively unchanged global gene expression profile, the cells were under translation stress, potentially stemming from disrupted transcription fidelity. Notably, we did not find any correlation between gene expression and pausing behavior–pausing of *dcr1*Δ-induced genes, for example, was not significantly altered in any of the mutants (Figure S2E). Altogether, this suggested that changes in nascent transcription dynamics were largely uncoupled from global steady-state gene expression profiles.

### Dcr1 cooperates with general transcription factors for pause release

We next investigated the role of Dcr1 with forward and reverse genetic analyses. Alleles of general transcription factors (GTFs) suppress pericentromeric heterochromatin silencing and/or quiescence defects of *dcr1*Δ, and we tested five of them (*tfs1*Δ, *mst2*Δ, fcp1-Y580*, *tbp1-D156Y*, and *med31-ins*) that regulate early transcription.^11,37,38^ All of them were able to suppress not only the TBZ sensitivity in *dcr1*Δ but also HU sensitivity in the triple mutant *dcr1*Δ *rnh1*Δ *rnh201*Δ (Figure S3A). Next, we obtained 14 HU-resistant suppressors in the triple mutant through an ethyl methanesulfonate (EMS) mutagenesis screen. To identify the candidate SNPs, we performed whole-genome sequencing and verified 8 by either backcrossing or re-introducing the SNPs (see STAR Methods; Table S4; Figure S3B–S3E and below). Notably, most replication stress suppressors also corresponded to GTFs regulating early transcription, strongly suggesting that T-R conflicts were induced in *dcr1*Δ.

One of the suppressors, tfa2-L238*, leads to the truncation of TFIIE-β, the smaller subunit of the transcription factor II E (TFIIE) complex.^39^ Although the truncated region is predicted to be highly disordered (Figure S3F), structural and biochemical studies with *S. cerevisiae* and human subunits suggested this region contacts the transcription factor II H (TFIIH) complex to stimulate promoter opening.^40,41^ Therefore, tfa2-L238* could have conferred HU resistance in the triple mutant by reducing the efficiency of RNA Pol II promoter escape. Interestingly, the single mutant tfa2-L238* was sensitive to TBZ (Figure S3E), suggesting a role in pericentromeric heterochromatin function. Indeed, pericentromeric transcripts *dg/dh* were derepressed in tfa2-L238* (Figure S3G), but not in *epl1-T449A*, another triple mutant suppressor that was insensitive to TBZ (Figure S3D).

**Figure 2.**
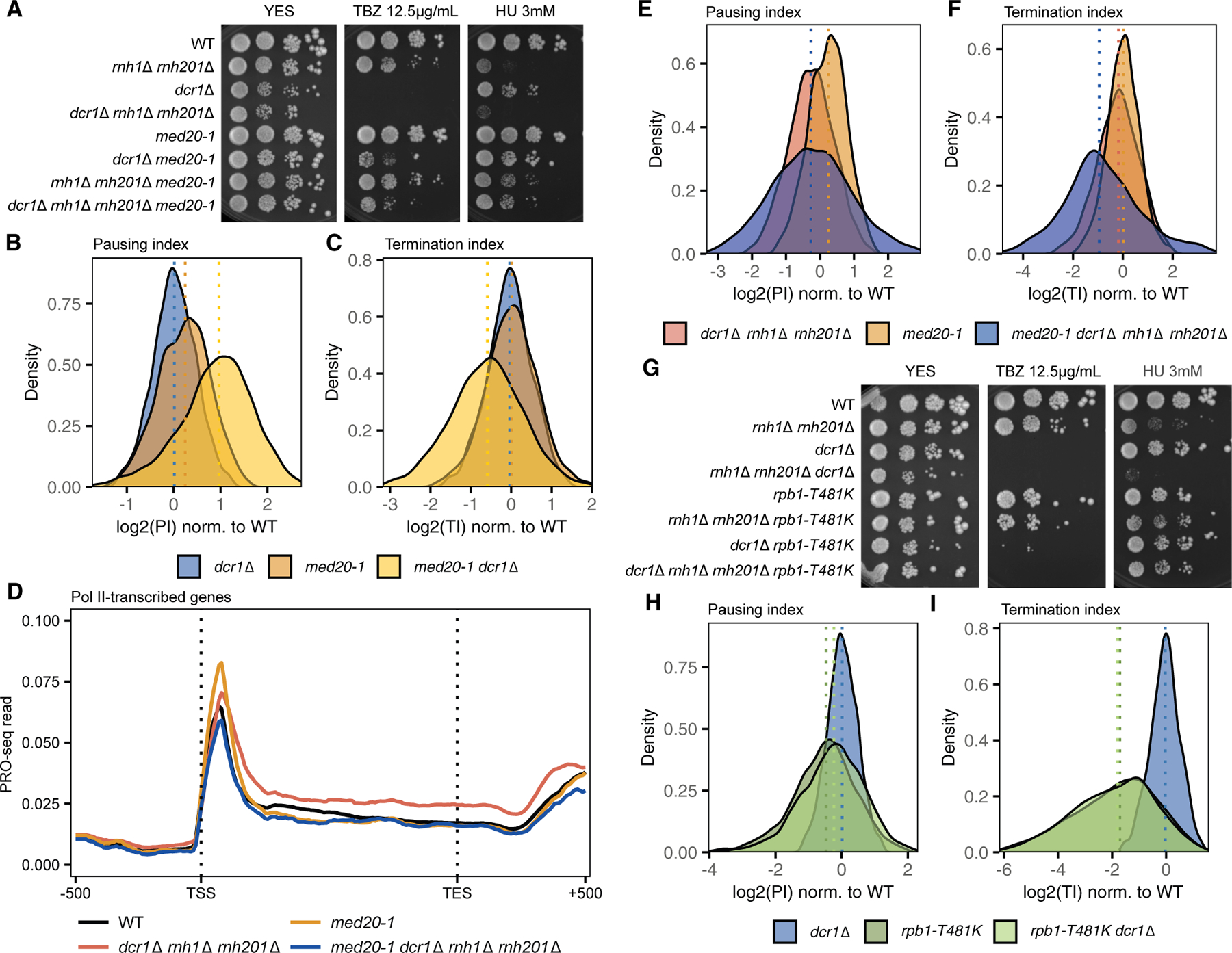
GTFs mediate R-loop-dependent replication stress (A) Spot growth assays of WT, *dcr1*Δ, *rnh1*Δ *rnh201*Δ, and the triple mutant *dcr1*Δ *rnh1*Δ *rnh201*Δ, with or without the mutant *med20–1* allele, spotted on YES plates without supplement or with TBZ or HU. (B and C) Density plot of pausing indices (PIs) (B) and termination indices (TIs) (C) in *dcr1Δ*, *med20–1*, and *dcr1Δ med20–1* after normalization to WT. Dotted lines represent the median for each genotype. (D) Detection of paused RNA polymerase by sequencing 3’-OH RNA ends using PRO-seq in asynchronized WT (black), *dcr1*Δ *rnh1*Δ *rnh201*Δ (red), *med20–1* (orange), and *med20–1 dcr1*Δ *rnh1*Δ *rnh201*Δ (blue) cells. Reads were normalized and mapped as in Figure 1H. (E and F) Density plot of PIs (E) and TIs (F) in *dcr1*Δ *rnh1*Δ *rnh201*Δ, *med20–1*, and *med20–1 dcr1*Δ *rnh1*Δ *rnh201*Δ after normalization to WT. Dotted lines represent the median for each genotype. (G) Spot growth assay of WT, *dcr1*Δ, *rnh1*Δ *rnh201*Δ, and the triple mutant *dcr1*Δ *rnh1*Δ *rnh201*Δ, with or without the mutant *rpb1-T481K* allele, spotted on YES plates without supplement or with TBZ or HU. (H and I) Density plot of PIs (H) and TIs (I) in *dcr1*Δ, *rpb1-T481K*, and *dcr1*Δ *rpb1-T481K* after normalization to WT. Dotted lines represent the median for each genotype.

Alleles of *med20* frequently appeared (3/14 suppressors) in our unbiased screen and were among the strongest suppressors of *dcr1*Δ (Figure S4). Med20 is part of the Med8/18/20 “movable jaw” subcomplex within the head module of Mediator,^42^ deletion of which compromises pericentromeric silencing.^43–45^ The three *med20* alleles we recovered, assigned *med20–1* to *med20–3*, disrupted conserved residues for *med20–1* (Y44S) and *med20–2* (W37A), or, in the case of *med20–3*, caused a frameshift at F108, leading to premature termination at position 128 and truncating roughly half of the protein (Figure S4A). We confirmed the suppressors by backcrossing and genotyping the *med20* mutants (Figures S4B–S4D). While both *med20–1* and *med20*Δ conferred HU resistance and none were TBZ-sensitive (Figures 2A and S4E), only *med20*Δ accumulated *dg/dh* pericentromeric transcripts (Figure S4F). In fact, *med20–1* suppressed silencing as well as the cellular quiescence phenotypes of *dcr1*Δ (Figures S4F and S4G,^11^). Thus, the roles of Med20 in pericentromeric silencing and in *dcr1*Δ-induced genome instability were functionally separable, implying the T-R stress-triggering loci lay outside the centromere. *med20–1* led to a global increase in pausing, which was dramatically elevated in *dcr1*Δ background (Figure 2B), concomitant with a drop in gene body reads and TI (Figures 2C and 2D). This suggests *med20–1* suppressed the *dcr1*Δ phenotype by limiting excessive RNA Pol II escape. Agreeing with this predicted function, *med20–1* also limited excessive gene-body readthrough in the triple mutant (Figures 2D–2F).

Two suppressors–*rpb1-T481K* and *rpb2-R1118H*–directly affected the large subunits of the RNA Pol II holoenzyme. Both alleles were sterile, and we verified their suppressibility by reintroduction into respective mutants (Figures 2G and S3B). The alleles disrupted highly conserved residues located close to each other within the catalytic center of RNA Pol II (Figure S5), with *rpb1-T481K* proximal to the NADFDGD motif that coordinates a Mg^2+^ ion for two-metal-ion catalysis,^46^ and *rpb2-R1118H* affecting the “switch 3” region that contacts the template DNA and the nascent RNA within the transcription bubble.^47^ We further studied *rpb1-T481K* by performing PRO-seq. While we observed no change to global median pausing level (Figure 2H), *rpb1-T481K* led to a dramatic decrease in TI, dominating the phenotype of *dcr1*Δ (Figure 2I). We speculate the threonine-to-lysine mutation displaces the catalytic triad, impairing RNA Pol II processivity in a manner similar to “slow” polymerase mutants.^48^ Proximity to the transcription bubble, where co-transcriptional R-loops are known to arise, is consistent with a role for D:R hybrids in Dcr1-dependent pause release.

### Dcr1’s helicase activity promotes genome maintenance

**Figure 3.**
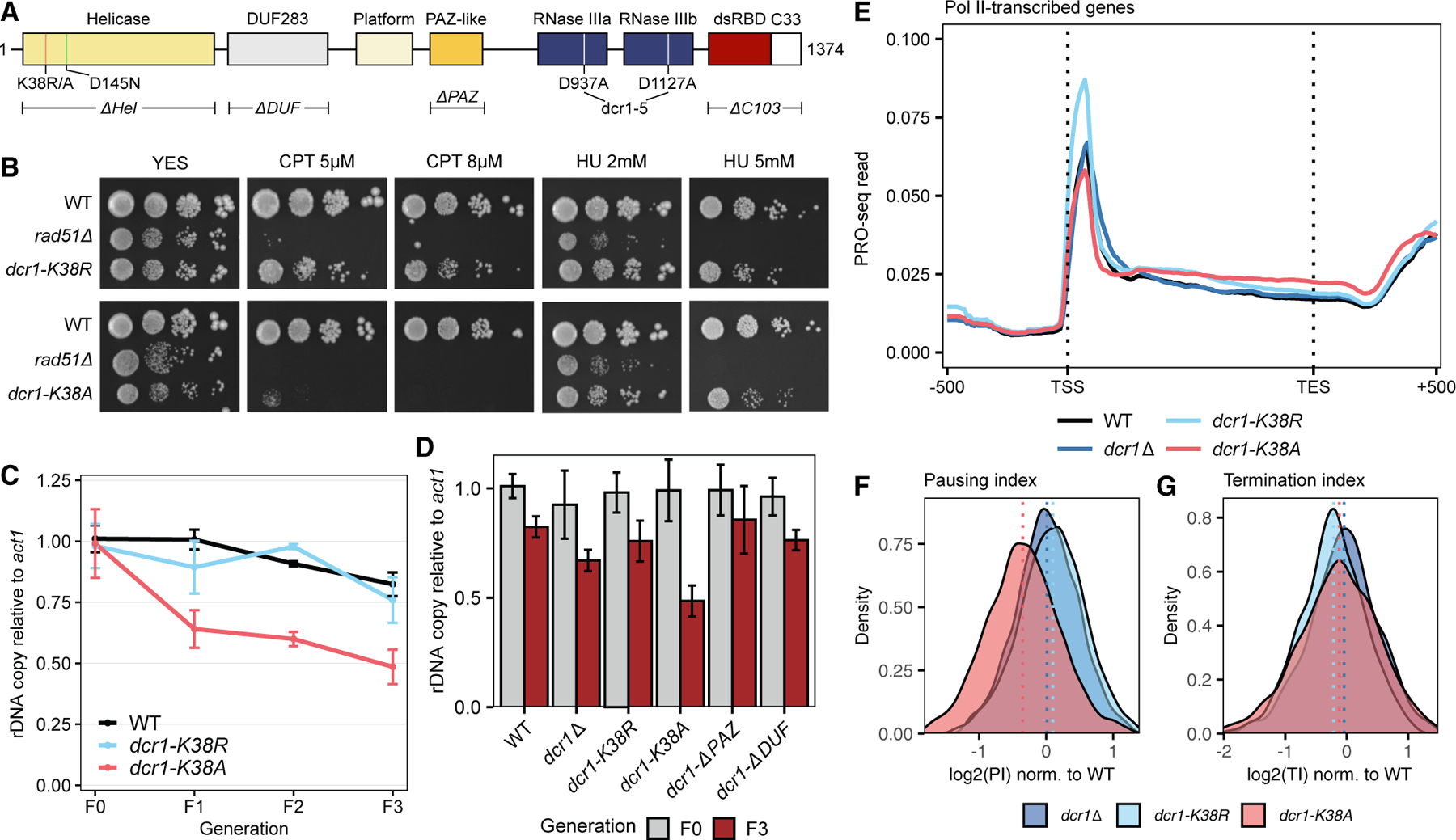
The helicase domain of Dicer is required for pausing and genome stability (A) Domain architecture of *S. pombe* Dcr1, highlighting the mutants used in this study. Not drawn to scale. (B) Spot growth assays of WT, *dcr1-K38R*, and *dcr1-K38A* on YES plates without supplement of with indicated concentrations of CPT and HU. *Rad51*Δ serves as a positive control. (C) qPCR quantification of relative rDNA copy number (WT F0 = 1) in WT, *dcr1-K38R*, and *dcr1-K38A* cells over 4 meiotic generations. (D) qPCR quantification of relative rDNA copy number (WT F0 = 1) in various *dcr1* alleles in F0 and F3 generation. (E) PRO-seq detection of paused RNA polymerase by sequencing 3^’^-OH RNA ends in asynchronized WT, *dcr1*Δ, *dcr1-K38R*, and *dcr1-K38A* cells. Reads were mapped and normalized as in Figure 1F. (F and G) Density plot of PIs (F) and TIIs (G) of all annotated genes in *dcr1*Δ, *dcr1-K38R*, and *dcr1-K38A* after normalization to WT based on PRO-seq data in (E). Dotted lines represent the median for each genotype.

Since previous studies showed that *dcr1–5*–an RNase III catalytic dead allele–had no defects in rDNA copy maintenance, unlike those in *dcr1*Δ,^4^ we performed a targeted domain analysis to uncover which part of Dcr1 was responsible for its genome maintenance phenotype. We deleted the Piwi/Argonaute/Zwille (PAZ)-like domain (Figure S6A), the DUF283 domain, the N-terminal helicase domain (ΔHel), and the C-terminal 103 amino acids (ΔC103) containing a dsRBD domain as well as a nuclear retention signal.^49^ We also generated *dcr1-K38R* and *dcr1-K38A*, disrupting the conserved DExD helicase Walker A motif^50^ (Figure 3A). As previously described,^51^ pericentromeric silencing was lost, and cells were TBZ-sensitive to varying degrees in all of the mutants (Figures S6B and S6C), indicating that an intact, functional Dcr1 was required for proper centromeric function. Unexpectedly, *dcr1-K38A*, but neither *dcr1-K38R* nor *dcr1-ΔHel*, caused CPT sensitivity (Figures 3B, S6C, and S6D). As the helicase deletion did not have a CPT phenotype, *dcr1-K38A* thus represented a dominant allele. This phenotype was additive with RNase H, as we found a negative interaction between *rnh1*Δ *rnh201*Δ and all the *dcr1* alleles tested (Figure S6E). We repeated the rDNA copy assay as previously described^4^ and found that only *dcr1-K38A*, but not *dcr1-K38R*, failed to maintain a WT rDNA copy level across meiotic generations (Figure 3C). Comparing rDNA copy number between F0 and F3, *dcr1-K38A* displayed the most severe loss of rDNA copies, even more than *dcr1*Δ (Figure 3D), again suggestive of a dominant phenotype. PRO-seq analyses revealed a pausing defect only in *dcr1-K38A* (Figures 3E–3G) and no significant termination defect (Figure 3F), phenocopying the triple mutant (Figures 1F–1H).

### Ago1 antagonizes Dcr1 in R-loop-induced genome instability

**Figure 4.**
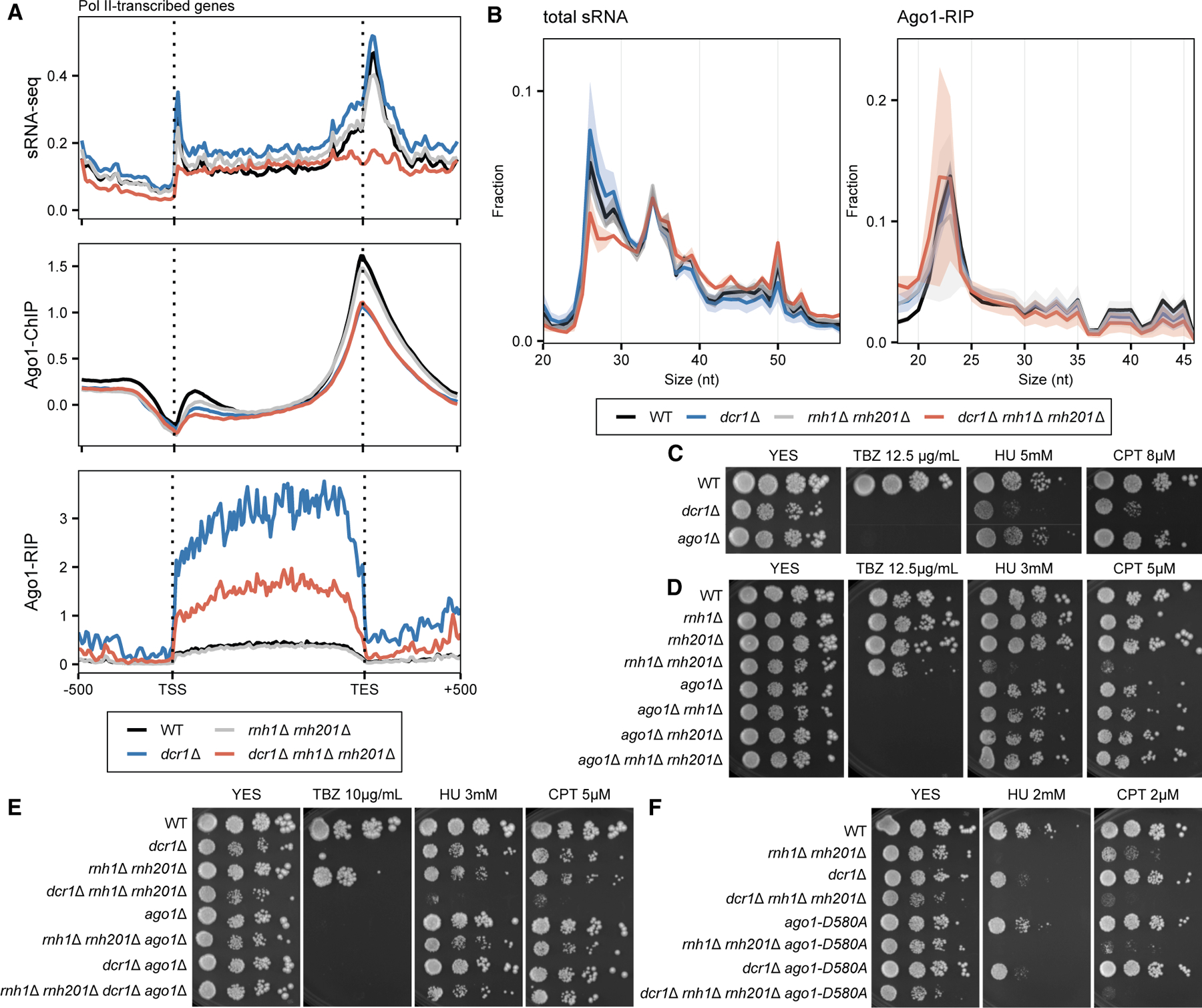
Dicer generates sRNAs resembling damage-associated sRNA (sdRNA) from R-loops that mediate replication stress via Ago1 (A) Detection of sRNA (top), chromosome-bound Ago1 (middle), and Ago1-associated sRNA (bottom) in WT (black), *dcr1*Δ (blue), *rnh1*Δ *rnh201*Δ (gray), and the triple mutant (red). sRNA, Ago1 ChIP, and Ago1 RIP sequencing reads were mapped to all annotated genes and normalized as in Figure 1F. (B) Size distribution of total sRNA (left) and Ago1-associated sRNA (right) in WT (black), *dcr1*Δ (blue), *rnh1*Δ *rnh201*Δ (gray), and the triple mutant (red). Sizes are indicated in nucleotides (nt). (C) Spot growth assays of WT, *dcr1*Δ, and *ago1*Δ cells with 10-fold dilution on YES plates without supplement or with indicated doses of TBZ, HU, or CPT. (D) Spot growth assays of WT and combinations of *rnh1*Δ, *rnh201*Δ, and *ago1*Δ cells with 10-fold dilution on YES plates without supplement or with indicated doses of TBZ, HU, and CPT. (E) Spot growth assays of WT and combinations of *rnh1*Δ, *rnh201*Δ, *ago1*Δ, and *dcr1*Δ cells with 10-fold dilution on YES plates without supplement or with indicated doses of TBZ, HU, and CPT. (F) Spot growth assays of WT and combinations of *rnh1*Δ, *rnh201*Δ, *ago1*-D580A, and *dcr1*Δ cells with 10-fold dilution on YES plates without supplement or with indicated doses of TBZ, HU, and CPT.

The genome stability function of Dcr1 is functionally separable from its role in sRNA-dependent pericentromeric silencing, as exemplified by the *epl1-T449A* and various *med20* alleles that suppressed one but not the other defect (Figures S3 and S4). Since Dcr1 is involved in sRNA biogenesis, we performed sRNA-seq to determine if RNase H loss affects the sRNA population. We detected a strong peak of sense strand sRNAs at TSS in WT that ranged predominantly between 25 and 40 nt (Figures 4A and 4B). The production of these promoter-derived sRNAs was redundantly dependent on both Dcr1 and RNase H, reflecting early transcription termination at pause sites. sRNA accumulation was also observed at the TES^4^ and also depended on both Dcr1 and RNase H (Figures 4A and 4B). However, we observed no substantial change in PRO-seq reads at termination sites (Figures 1H and 1J). Since PRO-seq only quantified transcriptionally productive RNA polymerases, RNA Pol II that accumulates downstream of TESs in *dcr1*Δ^4^ is not actively engaged in transcription and may undergo alternative mechanisms of termination.^52^

We next performed ChIP-seq of the sRNA-binding protein Ago1 to profile its binding pattern around euchromatic transcripts. The binding pattern of Ago1 largely followed that of sRNA and was reduced by sequentially deleting *dcr1*Δ and *rnh1*Δ *rnh201*Δ (Figure 4A). One possibility was that these sRNAs were loaded onto Ago1, which we tested by performing Ago1 RNA immunoprecipitation (RIP). Contrary to the TSS- and TES-peaks observed in sRNA-seq, Ago1-associated sense strand sRNA were found across the gene body and were greatly elevated in *dcr1*Δ cells (Figure 4A). These genic sRNAs were 20–25 nt in size, resembling centromeric small interfering RNA (siRNA) (Figure 4B), and were therefore distinct from the Dcr1-dependent promoter sRNA.

The observation that the Ago1 ChIP-seq profile largely matched that of sRNA, but that the sRNA was not loaded onto Ago1, as determined with RIP-seq, led us to hypothesize that Ago1 acted antagonistically to Dcr1 by binding to chromatin in a pathological manner during transcription-induced replication stress. Accordingly, in high doses of HU and CPT, only *dcr1*Δ but not *ago1*Δ displayed growth defects (Figure 4C). This prompted us to perform further genetic studies on *ago1*. We first tested whether *ago1* would genetically interact with RNase H in a similar way to that of *dcr1*. We observed no interaction between *ago1*Δ and *rnh1*Δ or *rnh201*Δ individually, but *ago1* deletion actually suppressed the HU and CPT sensitivity of the *rnh1*Δ *rnh201*Δ cells (Figure 4D), acting in the opposite way to *dcr1*Δ. These results were consistent with a previous proposal that Ago1 acted downstream of Dcr1 in the regulation of cell cycle checkpoint and cytokinesis.^8^ We therefore constructed combinations of mutants with *dcr1*Δ, *ago1*Δ, and *rnh1*Δ *rnh201*Δ. We observed no negative interaction between *dcr1*Δ and *ago1*Δ, as the double mutant appeared phenotypically identical to *dcr1*Δ. Strikingly, however, *ago1*Δ was able to suppress the ability of *dcr1*Δ to hypersensitize the RNase H mutant *rnh1*Δ *rnh201*Δ to both HU and CPT (Figure 4E). The results above suggested Ago1 was required for *dcr1*Δ to sensitize *rnh1*Δ *rnh201*Δ.

The fact that Ago1 remained largely bound to the transcription end site (TES) in the absence of sRNA (Figure 4A) suggested a role of Ago1 independent of its canonical RNAi function. Correspondingly, the slicer-defective Ago1-D580A failed to suppress the HU and CPT sensitivity of *dcr1*Δ and the triple mutant (Figure 4F). Therefore, promoter-proximal sRNA represents a distinct class of sRNA whose biogenesis depended on both Dcr1 and RNase H but was not bound to Ago1.

### Dcr1 limits genome instability in highly transcribed regions

**Figure 5.**
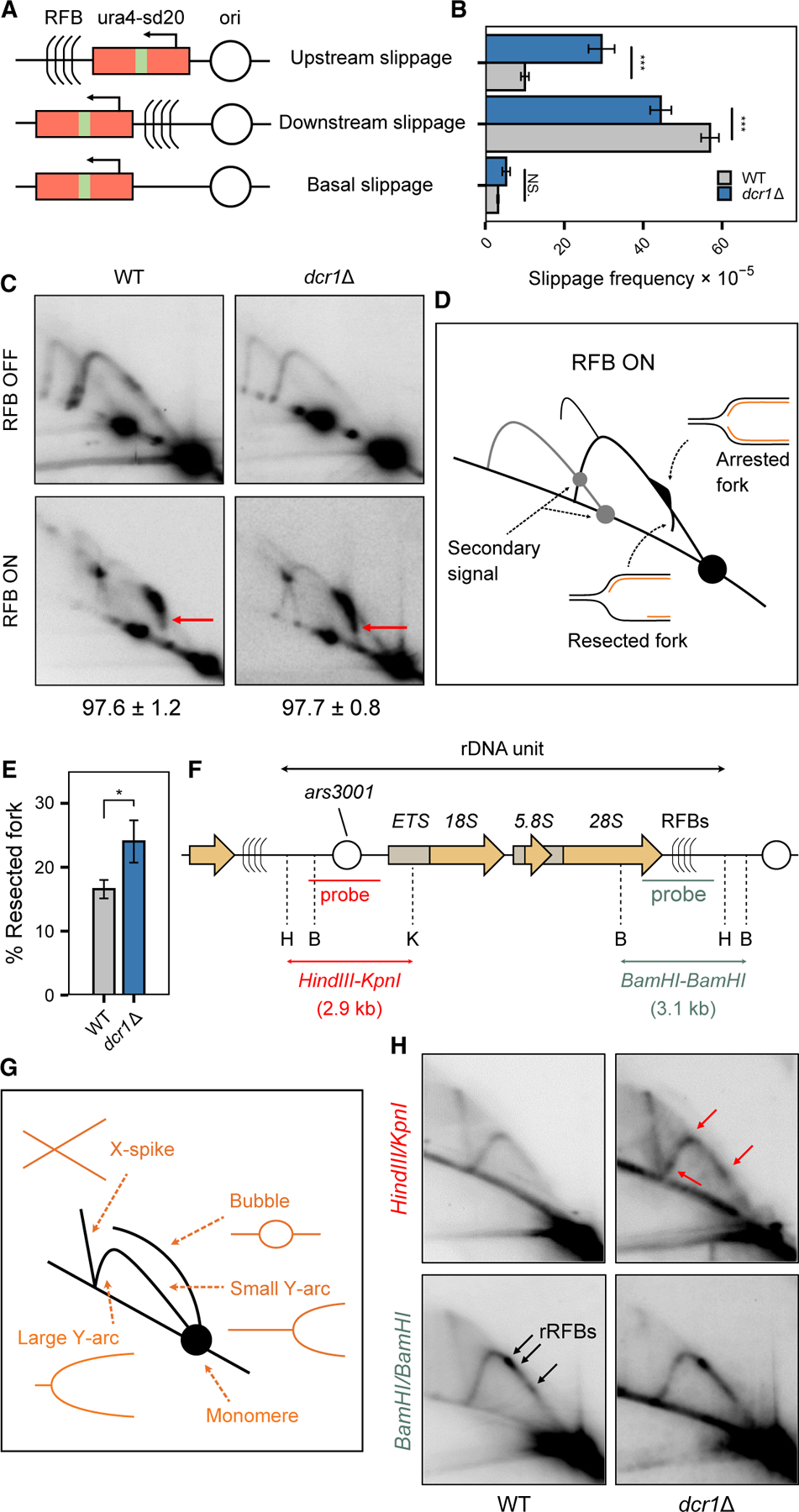
Dicer promotes faithful replication of highly transcribed genes near programmed replication fork blocks (A) Diagrams of constructs containing the reporter allele *ura4-sd20 (red rectangle)*, associated with the directional replication fork block *RTS1*-RFB from the rDNA locus on chromosome III. The RFB is integrated 5 kb away from a strong replication origin (ori). After thiamine removal, forks traveling from the centromere toward the telomere are blocked in a polar manner. The *ura4-sd20* allele contains a 20 nt duplication flanked by micro-homology. When the *ura4-sd20* allele is replicated by a restarted fork, the non-processive DNA synthesis undergoes RS, resulting in the deletion of the duplication and the restoration of a functional *ura4*^+^ gene. (B) Frequency of RS in indicated strains and constructs. Bars indicate mean values ± 95% confidence interval. Statistical analysis was performed using Student’s *t* test, compared with *WT*. *n* = 10–32. (C) 2D gel electrophoresis (2DGE) analysis of replication intermediates (RIs) in asynchronous WT and *dcr1*Δ cells using *ura4* as a probe. Representative gels are shown for thiamine-treated (RFB off) and untreated (RFB ON) cells undergoing slippage upstream of the RFB (A). The red arrow indicates the “tail” signal, which represents lagging-strand resection (D). Numbers indicate the efficiency of the RFB ± standard deviation (SD). (D) Schematics of RIs observed within the *AseI* restriction fragment in RFB ON condition. Gray lines indicate secondary signals caused by partial digestion of psoralen-crosslinked RIs. See (G) for annotation. (E) Tail quantification of resected forks from (C). Bars indicate mean values ± SEM. Statistical analysis was performed using Student’s *t* test, compared with WT. *n* = 2. (F) Schematic of an rDNA locus on chromosome III. Probes and restriction sites are indicated. H, *HindIII*; B, *BamHI*; K, *KpnI*; ars, autonomously replicating sequence; ETS, external transcribed spacer; RFB, programmed and polar RFB. The orange arrows indicate the transcription unit of rRNA. (G) Illustration representing the expected migration behavior of RI analyzed by 2DGE. The “Y arc” is a series of Y-shaped RI with progressively longer branches, resulting from replication fork progression within the DNA fragment analyzed. The “bubble arc” corresponds to the firing of the replication origin. The vertical X-spike results from X-shaped DNA joint molecules corresponding to RIs undergoing HR in *dcr1*Δ cells. (H) Representative images of RI analysis by 2DGE within the origin-containing *HindIII*-*KpnI* restriction fragment (top) and the RFB-containing *BamHI*-*BamHI* restriction fragment (bottom) in WT and *dcr1*Δ cells. Red arrows indicate fork pausing signals from T-R collisions on both sides of the origin in *dcr1*Δ cells, while black arrows indicate the position of the programmed RFBs in WT cells, respectively. *p < 0.05, ***p < 0.0005; NS, not significant.

All the evidence presented above suggested that Dicer modulates nascent transcription to limit T-R stress. We next investigated how replication was affected in the absence of Dcr1. We assessed how *dcr1*Δ affected fork progression with an engineered *RTS1*-replication fork barrier (RFB) fork stalling assay,^53^ in which Rtf1-bound *RTS1* blocked replisome progression in a polar manner, requiring rescue by RDR. Although the replication block is independent of transcription, accumulation of D:R hybrids has been reported in this system.^22,54^ RDR is prone to replication slippage (RS)^18^ and can be detected with *ura*^*+*^ reversion arising from the segmental-duplicated *ura4-sd20* allele (Figure 5A)^55^. The absence of Dcr1 resulted in a slight decrease (∼1.4-fold reduction) of RS downstream of RFB, occurring during replication restart (Figure 5B). By contrast, the frequency of upstream RS showed an ∼3-fold increase compared with WT. This may indicate that nascent strand degradation^18^ was more extensive, resulting in fork-restart downstream of the RFB occurring more often in *dcr1*Δ. Agreeing with this hypothesis, two-dimensional gel electrophoresis (2DGE) at the RFB (Figures 5C and 5D) revealed a strong signal corresponding to arrested forks at the *RTS1* barrier when RFB was activated. Exo1-mediated resected forks could be detected as a tail descending toward the linear arc,^56,57^ and the signal was more intense in *dcr1*Δ (Figure 5E).

We also analyzed replication fork progression within the highly transcribed rDNA repeats, which contained programmed RFBs and well-characterized replication origins (Figure 5F). 2DGE analysis revealed that, while both WT and *dcr1*Δ displayed the expected intensity along the Y arc corresponding to programmed fork pausing along the RFBs (Figures 5G and 5H), only *dcr1*Δ accumulated extra pausing signals in fragments containing the origin (Figure 5H, red arrows), as well as X-spike structures resembling recombination intermediates/joint molecules. These results suggested that replication was hampered in *dcr1*Δ when progressing through highly transcribed rDNA units, as previously observed at centromeres.^3^ Altogether, our data suggest Dicer prevents HR-mediated fork restart and potentially recombination at the rDNA by limiting nascent strand degradation.

**Figure 6.**
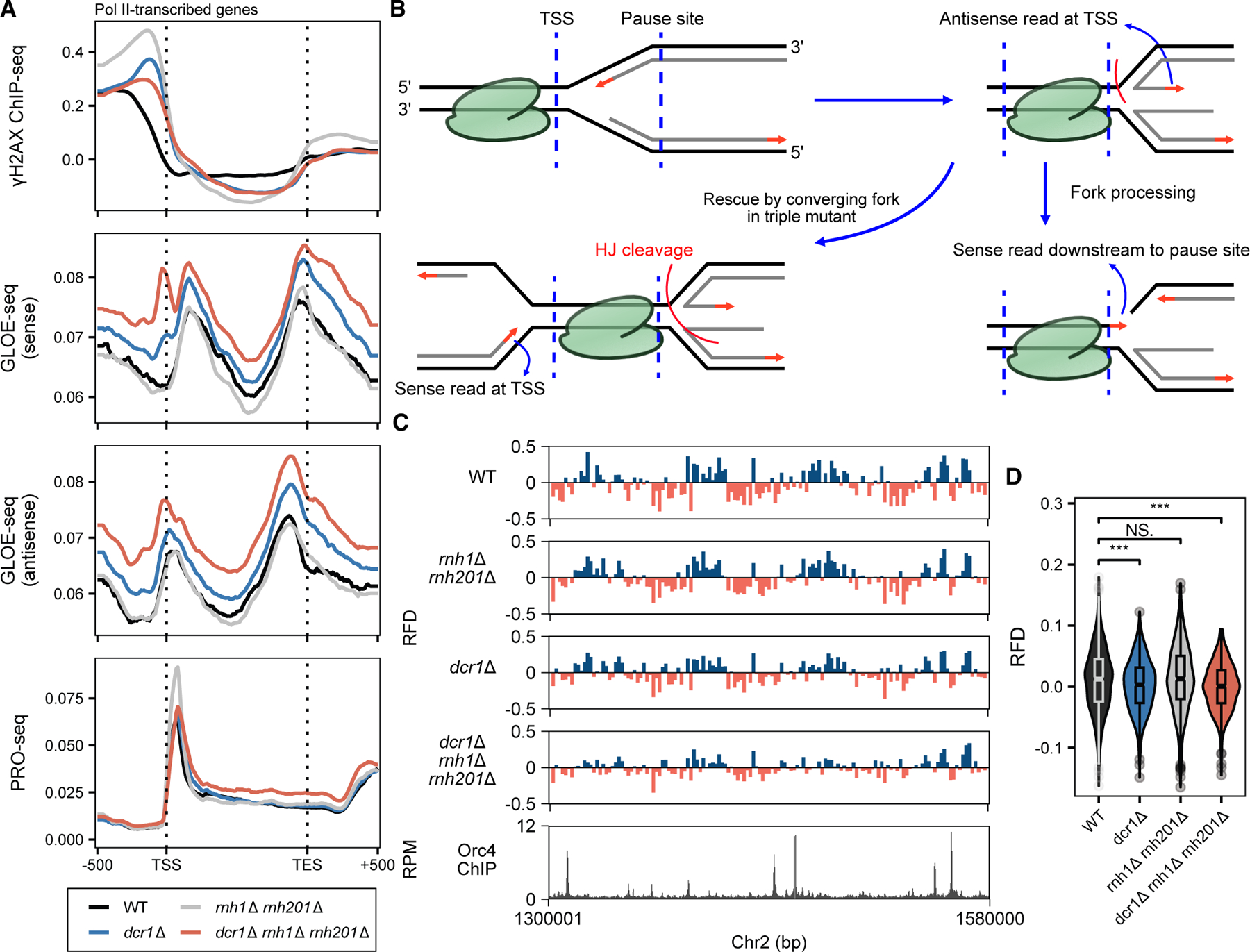
Dicer rescues stalled forks at R-loop-dependent T-R collisions (A) Detection of DNA damage, free 3^’^-OH ssDNA ends, and free 3^’^-OH RNA ends (paused RNA polymerase) in asynchronized WT (black), *dcr1*Δ (blue), *rnh1*Δ *rnh201*Δ (gray), and triple mutant (red) cells. From top to bottom: (upper) DNA damage detected by ChIP-seq of γH2A.X normalized to histone H2A, (middle) free 3^’^-OH single-strand DNA ends were detected by GLOE-seq from transcriptional sense and antisense strands, and (lower) 3^’^-OH RNA ends were detected by PRO-seq at transcriptional pause sites. GLOE-seq and PRO-seq read counts were mapped and normalized as in Figure 1F. (B) T-R collision at TSS, replication fork reversal at transcriptional pause sites, and rescue by converging forks are consistent with free ssDNA ends (sense peaks in A) arresting downstream of the pause site and leading strand DNA ends from convergent forks arresting at TSS in the triple mutant. Free ssDNA ends (antisense peaks in A) at the pause site in WT and at the TES in *dcr1*Δ and triple mutant cells correspond to leading strand ends arrested at T-R collisions. (C) Replication fork directionality (RFD) analysis of GLOE-seq data from (A). RFD is defined as the ratio of excess reverse (Crick strand, REV) reads within a region relative to forward (Watson strand, FWD) reads, which is calculated as (REV – FWD)/(REV + FWD). Replication origins were detected by Orc4 ChIP-seq^61^ (lower track) and correspond to leading to lagging-strand transitions. (D) Violin plot of the distribution of RFD at all annotated origins (pombase/oriDB), revealing significant replication fork asymmetry genome wide in *dcr1*Δ and triple mutant cells. *p* values represent results of one-way ANOVA. ***p < 0.0005; NS, not significant.

To investigate how Dicer prevents genome instability genome wide, we performed sequencing for DNA damage signatures. Phosphorylation of histone H2A (γH2AX) is one of the earliest responses in the face of DNA damage.^58^ Our results showed an enrichment of H2A phosphorylation upstream of TSSs in *dcr1*Δ, *rnh1*Δ *rnh201*Δ, and the triple mutant *dcr1*Δ *rnh1*Δ *rnh201*Δ (Figure 6A), indicating persistent DNA damage around promoters. To probe the nature of damage, we employed genome-wide ligation of 3’OH ends followed by sequencing (GLOE-seq),^59^ which detects free 3^’^-OH ssDNA ends, and aligned the reads sense or antisense to transcription. The triple mutant had a genome wide increase in free 3^’^-OH ssDNA ends around TSSs and TESs (Figure 6A), suggesting that paused RNA Pol II and unresolved R-loops led to DNA breaks. One possibility is that the breaks were linked to head-on T-R conflicts (Figure 6B): on the transcriptional antisense strand, free 3^’^-OH ssDNA ends accumulated at the TSS, consistent with leading strand termination at RNA Pol II initiation. On the transcriptional sense strand, free 3^’^-OH ssDNA ends peaked downstream of pause sites, but in the triple mutant, an additional peak accumulated immediately upstream of the TSS (Figure 6A). This could reflect processing of stalled or reversed forks by homologous recombination (HR) to remove paused RNA Pol II,^60^ resulting in nascent strand resection (Figure 5D), and rescue by converging forks (Figure 6B), respectively.

GLOE-seq also detects replication-dependent 3^’^-OH ends arising from Okazaki fragments.^59^ Changes to replication pattern could therefore be extrapolated by calculating the replication fork directionality (RFD) index, defined as the ratio of excess reverse strand (Crick strand) reads to the total amount of reads within a genomic region. We observed a clear genome-wide pattern of RFD that corresponded to Orc4 ChIP-seq peaks marking replication origins (Figure 6C). The bias was reduced in *dcr1*Δ and in the triple mutant (Figures 6C and 6D). While this could be due to an increase in leading or a reduction in lagging-strand reads, or a general decrease in replication speed, an interesting possibility is that replication forks, and associated breaks, were accumulating at promoters rather than at origins due to the presence of paused RNA Pol II and unresolved R-loops. This supports the idea that Dicer limits promoter-associated breaks by limiting fork processing and resection caused by roadblocks (Figure 5E).

### Dcr1 prevents head-on replication fork stalling

**Figure 7.**
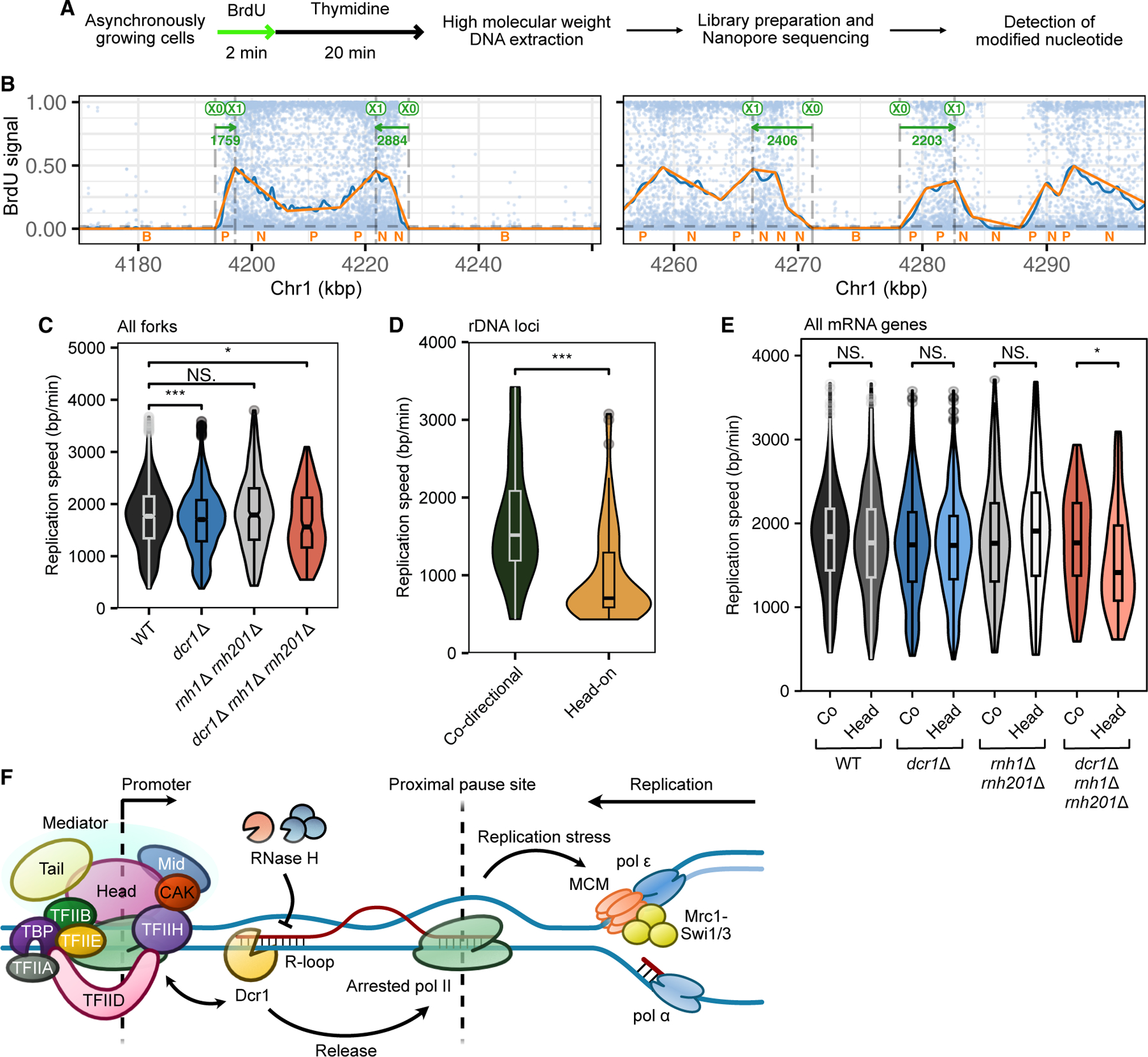
Dicer rescues head-on, but not co-directional, T-R collisions by processing R-loops (A) Schematic of pulse-chase labeling for replication fork detection by nanopore sequencing. (B) Example of individual nanopore sequencing reads from pulse-labeled WT (FY2317) cells, showing BrdU content after processing using NanoForkSpeed. Diverging forks (replication bubbles) appear as clusters of labeled nucleotides, with fork speed calculated by the slopes of relative signal density at the boundaries. Plots were generated using available dedicated software.^62^ (C) Violin plot of the global estimated replication speeds of forks detected in WT and mutant cells. *p* values represent results of one-way ANOVA. Fork progression is significantly slower in *dcr1*Δ and triple mutant cells. (D) Violin plot showing the estimated replication speeds for all replication forks mapped to the rDNA loci, separated according to head-on (Head) or co-directional (Co) with rRNA transcription. *p* values represent results of one-way ANOVA. (E) Violin plot of the estimated replication speed distributions for all replication forks near annotated mRNA transcripts, separated according to head-on or co-transcription to the direction of transcription direction, and by genotype. *p* values represent results of one-way ANOVA. (F) Model of promoter-proximal cleavage of R-loops by Dicer during T-R collisions. Most of the proteins indicated are required for silencing and/or negatively interact with Dicer during replication stress. *p < 0.05, ***p < 0.0005; NS, not significant.

In order to distinguish between these possibilities, we used nanopore long-read sequencing to estimate the replication speed of individual forks in a genome-wide manner.^62^ Here, an *S. pombe* strain capable of incorporating exogenous nucleosides^63^ was pulse-chased with bromodeoxyuridine (BrdU), and patterns of BrdU incorporation were detected and classified as replication forks using NanoForkSpeed^62^ (Figures 7A and 7B; see STAR Methods). In WT, we observed a median fork velocity of ∼1.8 kb/min in WT (Figure 7C), agreeing broadly with estimates in eukaryotes,^64^ though individual fork speed varied drastically, ranging from 0.5 to 3.6 kb/min (after removing the top and bottom 1% of outliers). We observed global changes to replication speed in the various mutant strains, with the median replication speed dropping by ∼0.1 kb in *dcr1*Δ and by another ∼0.15 kb in the triple mutant, to ∼1.55 kb/min (Figure 7C). Curiously, we observed a modest increase in speed in the *rnh1*Δ *rnh201*Δ mutants, which was surprising considering that R-loop accumulation is usually associated with impeded fork progression.

We segregated individual forks traversing co-directionally or head-on to annotated genes. In rDNA we observed a substantial reduction in velocity when the fork traveled head-on (Figure 7D), as expected. Strikingly, across all mRNA genes, we observed a similar significant reduction in replication speed as forks traveled head-on, but only in the triple mutant (Figure 7E). The fact that *dcr1*Δ led to a global slowdown of replication regardless of transcriptional directionality was consistent with the pause defect being pathological only when R-loops accumulated, as in the *rnh1*Δ *rnh201*Δ mutant. Therefore, Dcr1 promotes fork progression through head-on collisions with R-loops.

## DISCUSSION

Dicer is an ancient protein, believed to be present since the last eukaryotic common ancestor (LECA).^1,65^ Replication origins are often found near TSSs, generating T-R conflicts,^66^ and in *Saccharomyces sp.*, the emergence of sequence-specific replication origins correlates with the loss of RNAi, which is thought to increase T-R conflicts.^67^ Our finding in *S. pombe* supports this hypothesis, placing RNAi as a key player in resolving T-R conflicts at the promoter (Figure 7F): when RNA Pol II becomes pathologically arrested at TSSs–such as when R-loops accumulate–Dcr1 limits unscheduled pause release and is dependent on the catalytic activity of its helicase domain. The absence of this function leads to T-R conflicts and therefore replication stress. This was supported by three lines of evidence. First, Dcr1 and RNase H co-operated in sRNA production at the TSSs and TESs of expressed genes genome wide, corresponding to the location of R-loops that accumulated in RNase H mutants and are a signature of T-R stress (Figures 1F and 4A). Second, genetic studies (Figures 2, S3, and S4) identified multiple alleles of GTFs, strongly suggesting a link of *dcr1*Δ-induced T-R stress to early transcription. Third, PRO-seq analyses of nascent transcription dynamics revealed modifications in promoter-proximal pausing but relatively little change in termination behavior (Figures 1F–1H). This was further supported by *med20–1*, a Dcr1 suppressor, having dramatically enhanced pausing, especially in *dcr1*Δ (Figures 2B–2D).

In the triple mutant *dcr1*Δ *rnh1*Δ *rnh201*Δ, cells became elongated with multiple septa and nuclei (Figure 1D). One explanation for the replication phenotypes, therefore, could be defective cell cycle checkpoint enactment, potentially leading to mitotic progression before DNA synthesis was complete. This is indeed well documented in RNAi mutants,^8,68^ but this was unlikely to be the main cause for several reasons. First, while both *dcr1*Δ and *ago1*Δ are checkpoint defective,^8^ only *dcr1*Δ was sensitive to a high dose of genotoxic drugs (Figure 4D). Additionally, only *dcr1*Δ, but not *ago1*Δ, negatively interacted with *rnh1*Δ *rnh201*Δ (Figure 4E), ruling out the possibility that both performed identical functions in the same pathway. The fact that *ago1*Δ was able to partially suppress the triple mutant phenotype (Figure 4F) implicated Ago1 as being required for the *dcr1*Δ phenotype to manifest. We therefore concluded that defective checkpoint regulation alone was insufficient to explain the data presented in this study.

We note the parallels between Dcr1 and the Integrator complex, which was recently described to release RNA Pol II from euchromatic transcripts via endonucleolytic cleavage.^30–32^ First, human Integrator is recruited to promoter-proximally paused RNA Pol II,^69^ and DICER cooperates with BRCA1 and AGO1/2 to generate 20–35 nt damage-associated sRNA (sdRNA) at paused sites,^70^ similar to those reported here, suggesting DICER is also recruited to paused RNA Pol II. This is further supported by our observation that Dcr1 regulates pausing at RNA Pol II loci in the presence of R-loops. Second, Integrator is proposed to be recruited to promoter-proximal R-loops via its displaced ssDNA strand.^32^ Human DICER and *S. pombe* Dcr1 both bind R-loops^19^ (Figures 1A and S1C). Therefore, both Dicer and Integrator share common mechanisms in their recruitment. Third, Integrator terminates paused RNA Pol II via a cleavage mechanism, generating short RNA in the process.^31^ Our results indicate that promoter-proximal sRNA is dependent partly on a functional Dcr1, broadly agreeing with human DICER cleaving R-loops^19^ and generating sRNA at paused sites.^70^ Lastly, the RNA Pol II removal function of Integrator has been shown to limit R-loop-induced replication stress,^71^ parallel to our finding that Dcr1 reduces genotoxic stress in RNase H-deficient cells. As *S. pombe* lacks the Integrator complex, we propose Dcr1 in *S. pombe* is the functional equivalent of the Integrator complex in higher eukaryotes, terminating pathological RNA Pol II at the promoter and generating sRNA in the process. Indeed, in *C. elegans*, Integrator is implicated in the biogenesis of PIWI-interacting RNA (piRNA) by terminating promoter-proximally paused RNA Pol II,^72^ suggesting an intimate connection between early RNA polymerase termination and RNAi.

While we cannot rule out the participation of the RNase III domains in mediating T-R stress (Figure S6), the helicase domain appeared to play a more prominent role. For example, rDNA copy maintenance was independent of the catalytic activity of the RNase III domains^4^ but depended on a functional helicase (Figure 3B). Here, we discovered that mutation of *Dcr1-K38A*, but not *Dcr1-K38R*, resulted in additional genome instability phenotypes. Walker A lysine to arginine and alanine would abolish ATP hydrolysis and binding, respectively, as in the case of the Rad51 class recombinases.^73^ Indeed, *D. melanogaster* Dcr-2 displayed distinct nucleic acid metabolic activities when incubated with or without ATP or with the non-hydrolyzable ATPγS,^74^ suggesting that in Dicer too, ATP binding and hydrolysis were separable events, exerting different influences on Dicer activity.

We further characterized the consequences of genome instability in the absence of Dicer. In both *dcr1*Δ and *rnh1*Δ *rnh201*Δ, we observed accumulation of phospho-H2A around TSSs, but only in the triple mutant did it manifest as a detectable GLOE-seq peak representing DNA breaks (Figure 6A). This suggests that the absence of either Dcr1 or RNase H activates the DDR pathway, in agreement with phospho-H2A being one of the earliest damage markers, which does not necessarily mean the presence of a DNA break.^58,75^ From the GLOE-seq results, we also observed an altered RFD profile, suggesting replication-associated breaks accumulating at promoters rather than at origins. We demonstrated this with 2DGE assays as well as with single-molecule replication mapping using long-read sequencing. We showed a direct slowing down of replication as the fork progressed head-on to transcription units, supporting the role of Dcr1 in preventing T-R conflicts in the presence of R-loops.

We detected sRNA mapped to the TSSs and TESs from the transcribed strand, which depended redundantly on both Dcr1 and RNase H (Figure 4A). The absence of antisense sRNAs suggested that the sense strand sRNAs were cleavage products from nascent transcripts. Whether the sRNA serves a functional role remains unclear and could be the consequence of Dcr1 and RNase H terminating RNA Pol II via an endonucleolytic mechanism. As the resulting sRNA was not loaded onto Ago1, it was unlikely to serve to recruit Ago1 to TSS and TES. Instead, Ago1 has been hypothesized to be recruited via its binding to R-loops.^76^ In fact, the PIWI domain of Argonautes resembles an RNase H domain,^77^ and prokaryotic Argonaute proteins often display strong affinity for DNA substrates.^78^ Ago1 acts downstream of Dcr1,^8^ and we found that Ago1 was indeed recruited to TSSs, and especially TESs, by Dicer and RNase H (Figure 4A). As *ago1*Δ suppresses the genome instability phenotype of *dcr1*Δ (Figure 4F), we propose that aberrant recruitment of Ago1 to stalled RNA Pol II presents an obstacle to replication.

Recent evolutionary studies suggested that Argonaute and Dicer had distinct functions before being co-opted into silencing pathways.^79^ As descendants of Asgard Archaea,^80^ LECA very likely evolved with a pre-existing, functional Argonaute.^81^ In fact, Archaeal Argonaute has been found to participate in replication termination.^82^ Dicer, on the other hand, appeared to be a truly eukaryote-specific fusion protein with domains of bacterial and archaeal origins.^65^ Notably, the closest prokaryotic homolog to Dicer’s helicase is that of an archaeal protein, Hef,^65,83^ which participates in resolving stalled replication forks.^84^ The evolutionary origin of Argonautes and Dicer is still an active area of investigation, and a DExD domain helicase and RNase III-dsRBD chimeric gene have been found within “defense islands” containing RNA-guided Argonautes in Asgard Archaea,^85^ suggesting the emergence of a proto-RNAi pathway before LECA. Nevertheless, given the evolutionary context and the results presented here, we speculate the ancient function of Dicer and Argonaute predates the emergence of a silencing pathway and lies in limiting replication stress.

### Limitations of the study

While the principles of the mechanism by which Dicer impacts T-R collisions near the TSS are outlined in this study, a deeper understanding would be gained by further experimentation. For example, PRO-seq can only detect active RNA polymerase, while other forms of polymerase might also constitute replication barriers in the gene body. Similarly, R-loops that extend into the body of the gene require other detection techniques and could play an additional role to those found at promoters. Finally, we did not explore the role of Dicer in transcription termination, which is likely also important.

## RESOURCE AVAILABILITY

### Lead contact

Further information and requests for resources and reagents should be directed to the lead contact, Robert Martienssen (martiens@cshl.edu).

### Materials availability

Newly generated strains are available upon request and should be directed to the lead contact.

### Data and code availability

- All raw and processed data have been deposited at GEO under GEO: GSE278850. Raw imaging data have been deposited onto Mendeley data, accessible at doi: https://doi.org/10.17632/tbd9698xsj.1.
- While no new scripts were generated in this manuscript, pipelines used for downstream analyses and figure generation can be accessed at https://github.com/martienssenlab/R-loop-manuscript.
- Any additional information required to reanalyze the data reported in this paper is available from the lead contact upon request.

## STAR★METHODS

### EXPERIMENTAL MODEL AND STUDY PARTICIPANT DETAILS

The fission yeast *Schizosaccharomyces pombe* was used in this study. All strains used in this study are detailed in Table S1. All cultures were grown at 30°C in standard media with supplement (YES). Knockout mutants were generated following lithium-acetate transformation protocol^86^ with cassettes generated using primers listed in Table S2. Higher order mutants were either generated by further direct transformation or by crosses on ME plates.

### METHOD DETAILS

#### *S. pombe* growth and spot assays

For spot assays, mid-log phase cells were harvested and spotted with 10-fold serial dilutions. Cells were let grown for 3 – 5 days at 30°C before being photographed. For doubling time analyses, overnight cultures were diluted and let grown into early log phase, before O.D. measurements were taken every ~60 minutes. Doubling time was estimated by fitting the data with an exponential curve using a simple R script. Imaging was performed as previously described.^11^ Briefly, cells washed in 1X PBS were dried on positively charged slides, and stained with DAPI (Vector labs), and pictures were taken with an Axio Imager.M2 (Zeiss) microscope.

#### Purification of Dcr1

FLAG-Dcr1 strain, which was tested for functional silencing, was used for native purification. 5 L of YES medium was inoculated with 10 – 15 mL of saturated overnight culture and grown to O.D. of 1 – 1.5. Cells were spun at room temperature and washed with distilled water and frozen for storage at −80°C. The cell pellet was then resuspended in equal volumes lysis buffer (50 mM HEPES (pH 7.6), 300 mM potassium acetate, 5 mM magnesium acetate, 20 mM β-glycerol phosphate, 1 mM EGTA, 1 mM EDTA, 0.1% (v/v) NP-40) containing protease inhibitors. The suspension was aliquoted into 500 μL fractions in microcentrifuge tubes to which an equal amount of acid washed glass beads was added. The cells were disrupted in a FastPrep machine (4 rounds of disruption at 6 m/s). The cell lysate was then spun at 14,000 rpm for 15 min at 4 °C. The supernatant was transferred to a 50 mL Falcon tube and incubated with 500 μL of pre-washed anti-Flag-M2 agarose (Sigma) for 2 – 3 hrs at 4 °C. The beads and immobilized proteins were harvested by centrifugation at 500 × g, loaded on a Bio-Rad Polyprep column and washed three times with 10 ml lysis buffer minus proteases. Bound protein was eluted with 3 × 500 μL fractions with the same buffer containing 0.2 mg/mL 3 × Flag peptide (Sigma).

#### Electrophoretic mobility shift assay

Reaction mixtures (20 μL) consisted of 0.5 nM labelled synthetic nucleic acid substrate (Table S3) in Binding Buffer (50 mM Tris-HCl, pH 8.0, 1 mM DTT, 100 μg/mL BSA, 6% v/v glycerol). Reactions were started by adding proteins as indicated and held on ice for 15 min. The reaction mixtures were then loaded onto a pre-equilibrated 4 % native polyacrylamide gel in low ionic strength buffer (6.7 mM Tris-HCl pH 8.0), 3.3 mM sodium acetate, 2 mM EDTA pH 8.0). Samples were run in the gels at 200 V for 90 min with continuous buffer recirculation throughout. For all experiments, both buffer and gel were pre-cooled at 4 °C, and electrophoresis was done at room temperature. Gels were dried on 3 MM Whatman paper and exposed to phosphorimager screens overnight. Exposed screens were scanned using a Fuji FLA-3000 PhosphorImager. All the quantification was done with ImageQuant software (Fuji ImageGauge). All assays were repeated three times to ensure reproducibility.

#### Microscale thermophoresis

The sequences of oligonucleotides used are listed in Table S3. DNA was labeled using the 5’ EndTag nucleic acid labeling system (Vector laboratories, MB-9001), with Alexa Fluor™ 647 C_2_ Maleimide (ThermoFisher, A20347). D:R hybrid was annealed in a 20 μL reaction consisting of 125 ng of labeled DNA, an equimolar amount of RNA, 1.2 μL of 5M NaCl, and 16.8 μL 1M TE pH 7.0. 500 pM of D:R hybrid and 500 nM of Dicer was used for MST using the Monolith NT.1155 pico machine.

#### PRO-seq

PRO-seq libraries were performed as described,^87^ except cell permeabilization, run-on, and RNA extraction were performed following ref. Mahat et al.,^28^ to adapt for yeast cells. Briefly, cells were permeabilized and run-on was performed with 2-biotin, omitting biotin-11-ATP and biotin-11-GTP. Total RNA was extracted using hot phenol approach as described.^28^ The RNA was base hydrolyzed and excess unincorporated biotin-NTPs were removed before undergoing on-bead library prep as detailed in ref. Judd et al.^87^ The libraries were sequenced on Illumina NextSeq platform.

#### Analysis of RNA by RNA-seq and RT-qPCR

Total RNA was purified from mid-log phase cells using Direct-zol RNA Miniprep Kit (Zymo Research) following manufacturer’s instructions, including the use of ZR BashingBead Lysis Tubes to lyse open the cells in TRI Reagent. DNA was removed using DNase I (Zymo Research) and purified using RNA Clean & Concentrator (Zymo Research).

For RT-qPCR, cDNA was generated from 1 μg of total RNA using SuperScript IV Reverse Transcriptase (Invitrogen 18090010) and random hexamer following the manufacturer’s instruction. The cDNA was diluted 1:20 and 2 μL of diluted cDNA was used per reaction. PowerUp SYBR Green Master Mix (Applied Biosystems) was used and measurements were taken using QuantStudio 5 or 6 machines (Applied Biosystems). The runs were analyzed with the ΔΔCt method, with *act1* as control.

For RNA-seq, Ribosomal RNAs were depleted using RiboMinus Transcriptome Isolation Kit, Yeast (Invitrogen), and libraries were made using NEBNext Ultra II Directional RNA Library Prep Kit (NEB). The barcoded multiplex libraries were pooled and sequenced on Illumina NextSeq platform.

#### sRNA-seq and Ago1 RIP-seq

Total RNA was purified as described above, and libraries were prepared using NextFlex Small RNA-seq v3 Kits (PerkinElmer) and sequenced on NextSeq Illumina platform. Reads were first filtered and trimmed using fastp.^88^ Duplicated reads and reads smaller than 20 nt or larger than 70 nt were discarded. Reads were mapped mapped to the *S. pombe* genome using Bowtie2,^89^ and normalized to RPM before analysis using custom R scripts.

For Ago1 RIP-seq, the starting material was immunoprecipitated sample following the ChIP protocol described below. Samples were de-crosslinked and treated with RQ1 DNase (Promega). Libraries were prepared using NextFlex Small RNA-seq v4 Kits (Revvity). Reads were processed the same way as sRNA-seq.

#### Suppressor screen

EMS mutagenesis was carried out as previously described.^90^ Briefly, 1 × 10^9^ the triple mutant yeast cells were harvested at mid-log phase, washed with sterile water and resuspended in 1.5 ml of 0.1 M sodium phosphate buffer (pH 7.0) and treated with 50 μL of EMS (Sigma M0880) for 1 h, 30 °C. 0.2 mL of treated cells was moved to 8 mL of sterile 5% sodium thiosulfate to inactivate the EMS. Cells were washed, resuspended in sterile water and plated on YES plate supplemented with 5 mM HU. Single colonies were allowed to recover on YES plate before re-streaking to HU plates to verify suppression. For whole-genome sequencing, genomic DNA was purified using Quick-DNA Fungal/Bacterial Kits (ZymoResearch). DNA library was constructed NebNext Ultra II DNA Library Prep Kit for Illumina (NEB E7645). Barcoded DNA library was sequenced using Illumina NextSeq500 Paired-End 76 bp and analyzed as previously described.^11^ Briefly, reads were adapter-trimmed and quality-filtered using Sickle (paired-end mode), then mapped to the *S. pombe* genome using Bowtie2.^89^ Duplicate reads were removed using Samtools, and SNPs were called usingayes to find SNPs present in suppressor strain but absent in the parental strain.^91^

#### Chromatin immunoprecipitation (ChIP) and ChIP-seq

ChIP was performed as described^92^ with slight modifications. Briefly, 40 mL of mid-log phase cells in YES were crosslinked with 1% formaldehyde for 20 minutes and quenched with 360 mM glycine and 2.4 mM Tris for 5 minutes. Whole cell extracts were then prepared with FastPrep-24 and the chromatin was sheared with BioRuptor using 15 cycles of 30s ON/30s OFF. 2 μg of antibodies were pre-conjugated to 15 μL of Pierce Protein A/G Magnetic Beads (Thermo Scientific) for ∼3 hours at 4 °C before being added to the sheared chromatin and were incubated overnight at 4 °C. The beads were washed and de-crosslinked with proteinase K and overnight incubation at 65°C. ChIP DNA Clean & Concentrator (Zymo Research) was used to clean up the DNA, and libraries were prepared using NEBNext Ultra II DNA Library Prep Kit (NEB). Indexed libraries were sequenced on NextSeq Illumina platform. For data processing, the demultiplexed reads were first trimmed with cutadapt^93^ before mapping to the genome using Bowtie2.^89^ Reads were converted to sorted bam files usins,^94^ which were then used to generate normalized tracks using deepTools^95^ and further analyzed using custom R scripts.

For, dRNH1 ChIP-seq the strains and dRNH1 induction was followed using the method described in Sagi et al.,^20^ but the subsequent chromatin extraction, immunoprecipitation, and library preparation was performed following the steps described above for consistency.

#### GLOE-seq

GLOE-seq libraries were prepared as described^59^ except Zymolyase 100T was used instead of 20T for a more efficient spheroplasting. Data analyses were performed using a custom R script that is accessible on https://github.com/martienssenlab/R-loop-manuscript.

#### Long-read sequencing analysis of replication

For replication analysis, the strain FY2317 (from Forsburg lab) carrying the *hsv-tk* and *hENT1* transgenes^63^ was used for pulse-chase experiments. Additional mutants were created from FY2317 specifically for replication analyses (Table S1). All strains were tested for 5-fluoro-2’-deoxyuridine (FuDR, Sigma F0503) sensitivity and dot blots were performed to confirm capability of BrdU incorporation (data not shown). The experimental procedure of pulse-chasing experiment largely followed that described in ref. Theulo et al.,^62^ and DNA extraction from ref. Hennion et al.,^96^ with slight modification. Briefly, 650 μM of BrdU was added to 50 mL of mid-log phase cells for 2 minutes, before being chased with 6.5 mM of thymidine for 20 minutes. The cells were then pelleted, washed twice with water, before being spheroplasted with 125 μL of 50 mg/mL zymolyase 100T in 2 mL of Y1 buffer (1M sorbitol, 100 mM EDTA, pH 8.0, 14 mM β-mercaptoethanol), for ∼30 minutes at 30 °C or until > 90% cell lysis when made 1% with SDS. Spheroplasts were then pelleted and lysed in 500 μL of 10% SDS, and incubated with 15 μL RNase A (Thermo Scientific) for 30 minutes at 50 °C. The lysate was cooled on ice, and 10 mL of TE and 5 mL of 5M potassium acetate was added. After 10 minutes on ice, the lysate was cleared by centrifuging at 5,000 x g at 4 °C for 10 minutes. 1 volume of isopropanol was added to precipitate the DNA, and was hooked using bent glass tip and washed twice with 70% ethanol. The hmwDNA was resuspended by incubating in 100 μL of TE at 50 °C for 30 minutes and its integrity confirmed by gel electrophoresis. 2 μg of hmwDNA was gently sheared by slowly pipetting up and down with a p200 tip, and library was prepared using ligation sequence kit SQK-LSK109 (ONT) and sequenced using R9.4.1 chemistry flow cells. Fork detection and replication speed analyses were done using NanoForkSpeed.^62^

#### Replication slippage assay

5-FOA (EUROMEDEX, 1555) resistant colonies were grown on uracil-containing plates with or without thiamine for 2 days at 30 °C. They were subsequently inoculated into EMMg supplemented with uracil, with or without thiamine, for 24 hours. Cells were diluted and plated on EMMg complete (for cell survival) and on EMMg uracil-free plates, both supplemented with 60 μM thiamine. Plates were incubated at 30 °C for 5 to 7 days. The reversion frequency was calculated as a ratio of the number of colonies grown on uracil-free plates (Ura+ colonies) to the number of viable cells plated.^55^

#### Bi-dimensional gel electrophoresis

For analysis of fork progression within rDNA, 2 × 10^9^ exponentially growing cells were mixed with 50 ml of frozen Azide-Stop solution (0.5 M NaOH, 0.4 M EDTA, 0.2% sodium azide) and spun down at 1,521 x g, 10 min at 4 °C. The pellet was washed in 20 mL cold water and spun at 3,434 x g, 5 min at 4 °C. Cells were resuspended in 5 mL NIB buffer (17% glycerol, 50 mM MOPS pH 7.2, 150 mM potassium acetate, 2 mM magnesium chloride, 500 μM spermidine and 150 μM spermine, 25 mg/ml of Lysing enzyme and 10 mg/mL of zymolyase 100T) and incubated at 37 °C for 15 min. Four volumes of water were added and cells were spun down at 3,434 x g, 5 min at 4 °C. 5 mL of G2 buffer (Genomic DNA buffer set, Qiagen) was added to the pellet and cells were gently resuspended and incubated with 200 μL of 10 mg/mL RNase A, 200 μl of 20 mg/mL proteinase K, and 1.5% lauroylsarcosine, at 50 °C for 1 hour. Cells were spun down at 3,434 x g, 5 min at 4 °C. The supernatant was further incubated with 100 μL of RNase A, 100 μL of proteinase K for 1 hour at 37°C, whereas the pellet was resuspended in 5 mL of Buffer G2 with 100 μL of RNase A, 100 μL of proteinase K, 1.5% lauroylsarcosine and incubated at 37 °C for 1 hour. Both the “pellet” and “supernatant” fractions were spun down at 3,434 x g, 5 min at 4 °C and supernatants were transferred onto Genomic-tip 100/g columns (Qiagen) according to the manufacturer instructions. DNA was eluted using 50 °C pre-warmed elution buffer and precipitated with isopropanol. 5 μg of genomic DNA was digested with either 60 Units of *BamHI* or 60 Units of *HindIII* and *KpnI* enzymes. Precipitated DNA was run on 0.4% agarose gel for the first dimension and a 1% agarose gel for the second dimension. DNA was capillary transferred in 10X SSC onto nylon membranes and probed with a radio-labeled ^32^P DNA probe corresponding to the 1,354 bp *EcoRI*-*EcoRI* fragment from rDNA unit (obtained by PCR using the following primers: GAATTCGGTAAGCGTTGGATTG and GAATTCTTCTTTCACATCTCC) for *BamHI*-digested sample or corresponding to 1,340 bp fragment of rDNA (obtained by PCR using the following primers: CATGGTTACGGTTACATTGG and CCATCCCATATTTCGCACGA) for *HindII*-*KpnI*-digested samples. Quantitative densitometry analysis of the resulted Southern-blots was carried using a phosphor-imager (Typhoon-trio) and ImageQuant software (GE healthcare).

For replication analysis at the *RTS1*-RFB locus, 2.5 × 10^9^ exponentially growing cells were treated with 0.1% sodium azide and mixed with frozen EDTA. Genomic DNA was crosslinked upon trimethyl psoralen (0.01 mg/mL, TMP, Sigma, T6137) addition to cell suspensions, for 5 min in the dark with occasional swirling. Cells were then irradiated with UV-A (365 nm) for 90 s at a constant flow of 50 mW/cm^2^.^97^ Cell lysis was performed using 0.625 mg/mL lysing enzymes (Sigma, L1412) and 0.5 mg/mL zymolyase 100 T (Amsbio, 120493–1). Resulting spheroplasts were embedded into 2% low-melting agarose (Lonza, 50081) plugs. Next, plugs were incubated overnight at 55 °C, in a digestion buffer with 1 mg/mL of proteinase K (Euromedex, EU0090), prior to washing and storage in TE buffer (50 mM Tris, 10 mM EDTA) at 4 °C. DNA digestion was performed using 60 units per plug of restriction enzyme *Ase*I (NEB, R0526M). Samples were treated with beta-agarase (NEB, M0392L) and RNase A (Roche, 11119915001) and equilibrated to 0.3 M NaCl, and then loaded onto BND cellulose columns (Sigma, B6385) to purify replication intermediates (RIs).^98^ Briefly, BND cellulose was dissolved in 0.3 M NaCl, 10 mM Tris-HCl (pH 7.5), 1 mM EDTA and packed into columns (Bio-Rad, 731–1550). Double-stranded DNA (dsDNA) was eluted by washing with 0.8 M NaCl, 10mM Tris-HCl (pH 7.5), and 1 mM EDTA. DNA containing single-stranded regions (ssDNA), such as RIs, was eluted by addition of 3 ml of 1 M NaCl, 10 mM Tris-HCl (pH 7.5), 1 mM EDTA, and 1.8% caffeine (Sigma, C-8960). RIs were precipitated with glycogen (Roche, 1090139001) and then separated by two-dimensional electrophoresis using 0.35 % and 0.9 % (+EtBr) agarose gels (1xTBE) for the first and second dimensions, respectively. Migrated DNA was transferred to a nylon membrane (Perkin-Elmer, NEF988001PK) in 10x SSC and probed with ^32^P-radiolabeled *ura4* probe (TaKaRa *Bca*BEST™ Labeling Kit, 6046 and alpha-^32^P dCTP, Perkin-Elmer, BLU013Z250UC) in Ultra-Hyb buffer (Invitrogen, AM8669) at 42 °C. Signal of replication intermediates was collected in phosphor-imager software (Typhoon-trio) and quantified by densitometric analysis with ImageQuantTL software (GE healthcare). The ‘tail signal’ was normalized to the overall signal corresponding to arrested forks.

### QUANTIFICATION AND STATISTICAL ANALYSIS

#### PRO-seq

Raw Fastq reads were processed and filtered using fastp,^88^ mapped with Bowtie2,^89^ and clipped to the 5’ position using Bedtools^99^ to map the position of Pol II at nucleotide resolution. The resulting BedGraph file was normalized by the total amount of reads and converted to BigWig format before being analyzed on R using custom scripts. Pausing indices (PIs) and termination indices (TIs) were calculated as previously described.^33^ For the calculation of TIs, to minimize interference from transcription from downstream genes, only genes that were at least 500 bp away from another annotation on the same strand were considered, resulting in a gene set of n = 1,299 genes.

Statistical analyses for PIs and TIs were carried out in R using one-way ANOVA, with the respective *p* values indicated on the graphs. NS. indicates *p* values > 0.05.

#### RNA-seq

The raw Fastq reads were trimmed using cutadapt^93^ and quantified using Salmon,^100^ using transcriptome annotation data from PomBase. Downstream data analyses were performed on R using custom scripts and differentially expressed genes called with DE-Seq2.^101^ Individual mutants were compared to WT for up- or down-regulated genes, defined as log_2_ fold-change (FC) > 2 and adjusted *p* value < 0.05. To look for genes specifically up-regulated in the triple mutant, *dcr1*Δ was classified as a ‘condition’, and WT and *rnh1*Δ *rnh201*Δ were classified as genotypes for gene-condition analysis. In this case, a less stringent criteria of log_2_ FC > 0 was used. See associated scripts deposited onto GitHub for details.

#### Statistical analysis

Student’s *t*-tests were used for the replication slippage and end resection assays. One way ANOVA was used for global RFD profiles and replication fork speed measurements, and Paired Wilcoxon tests were used for co-directional vs. head-on replication speed analyses. Respective *p* values were reported in the figure legends.

## Supplementary Material

MMC3

MMC2

MMC1

Supplemental information can be found online at https://doi.org/10.1016/j.molcel.2025.10.003.

## Figures and Tables

**Table:** KEY RESOURCES TABLE

REAGENT or RESOURCE	SOURCE	IDENTIFIER
Antibodies		

Anti-Histone H2A antibody	Active Motif	Cat. No. 39945; RRID: AB_2793402
Anti-Histone H2A (phosphor S129)	Abcam	Cat. No. ab15083; RRID: AB_301630
Anti-RNA polymerase II CTD repeat YSPTSPS (phospho S2)	Abcam	Cat. No. ab5095; RRID: AB_304749
Anti-RNA polymerase II CTD repeat YSPTSPS (phospho S5)	Abcam	Cat. No. ab5131; RRID: AB_449369
Flag-M2 (mouse)	Sigma-Aldrich	Cat no. F3165; RRID: AB_259259

Chemicals, peptides, and recombinant proteins		

3x FLAG peptide	Sigma-Aldrich	Cat. No. F4799
5-Fluoroorotic acid (5-FOA): Replication slippage assay	Euromedex	Cat. No. 1555
5-Fluoroorotic acid (5-FOA): All others	Thermo Scientific	Cat. No. R0811
5-Fluoro-2’-deoxyuridine (FuDR)	Sigma-Aldrich	Cat. No. F0503
5-Bromo-2’-deoxyuridine (BrdU)	Sigma-Aldrich	Cat. No. B5002
Anti-FLAG M2 Agarose	Sigma-Aldrich	Cat. No. A2220
Alexa Fluor™ 647 C_2_ Maleimide	Invitrogen	Cat. No. A20347
AMPure XP beads	Beckman Coulter	Cat. No. A63881
Benzoylated Naphthoylated DEAE-Cellulose	Sigma-Aldrich	Cat. No. B6385
Beta-agarase	NEB	Cat. No. M0392L
Biotin-11-CTP	Jena Bioscience	Cat. No. NU-831-BIOX
Biotin-11-UTP	Jena Bioscience	Cat. No. NU-821-BIOX
Caffeine	Sigma-Aldrich	Cat. No. C8960
Camptothecin	Sigma-Aldrich	Cat. No. C9911
cOmplete, Mini, EDTA-free protease inhibitor cocktail	Roche	Cat. No. 11836170001
DNase I	Zymo Research	Cat. No. E1011
Ethyl methanesulfonate	Sigma-Aldrich	Cat. No. M0880
Formaldehyde	Cell Signaling Technology	Cat. No. 12606
Glycogen	Roche	Cat. No. 1090139001
Hydroxyurea	Sigma-Aldrich	Cat. No. H8627
Lysing enzymes	Sigma-Aldrich	Cat. No. L1412
Pierce Protein A/G Magnetic Beads	Thermo Scientific	Cat. No. 88803
RNase A: 2DGE	Roche	Cat. No. 11119915001
RNase A: All other experiments	Thermo Scientific	Cat. No. EN0531
RQ1 DNase	Promega	Cat. No. M6101
SuperScript IV First-Strand Synthesis System	Invitrogen	Cat. No. 18091050
Thiabendazole	Sigma-Aldrich	Cat. No. T8904
Zymolyase 100T	Amsbio	Cat. No. 120493–1

Critical commercial assays		

5’ EndTag nucleic acid labeling system	Vector Laboratories	Cat. No. MB-9001
ChIP DNA Clean & Concentrator	Zymo Research	Cat. No. D5205
Direct-zol RNA Miniprep Kit	Zymo Research	Cat. No. R2051
Ligation Sequencing Kit	Oxford Nanopore Technologies	Cat. No. SQK-LSK009
PowerUp SYBR Green Master Mix	Applied Biosystems	Cat. No. A25742
RiboMinus Transcriptome Isolation Kit, Yeast	Invitrogen	Cat. No. K155003
NEBNext Ultra II Directional RNA Library Prep Kit	NEB	Cat. No. E7760
NEBNext Ultra II DNA Library Prep Kit for illumina	NEB	Cat. No. E7645
NextFlex Small RNA-seq v3	Bioo Scientific	Cat. No. 5132–06
NextFlex Small RNA-seq v4	Revvity	Cat. No. NOVA-5132–31

Deposited data		

Raw and analyzed data	This study	GEO: GSE278850
Raw images	This study	doi: https://doi.org/10.17632/tbd9698xsj.1

Experimental models: Organisms/strains		

*S. pombe* strains	This study	Table S1

Oligonucleotides		

Oligonucleotides: *S. pombe* strains related	This study	Table S2
Oligonucleotides: Microscale thermophoresis	This study	Table S3

Software and algorithms		

BedTools v2.29.2	Masuda et al.^61^	https://bedtools.readthedocs.io/en/latest/
Bowtie2 v2.4.2	Murray et al.^86^	https://github.com/BenLangmead/bowtie2
ChimeraX v1.8	Judd et al.^87^	https://www.rbvi.ucsf.edu/chimerax/
Cutadapt v4.4	Chen et al.^88^	https://cutadapt.readthedocs.io/en/stable/
DeepTools v.3.5.0	Langmead and Salzberg^89^	https://deeptools.readthedocs.io/en/develop/
DESeq2 v1.30.1	Winston^90^	https://bioconductor.org/packages/devel/bioc/html/DESeq2.html
Fastp v0.23.4	Garrison and Marth^91^	https://github.com/OpenGene/fastp
FreeBayes v1.1	Kim et al.^92^	https://github.com/freebayes/freebayes
Salmon v1.5.1	Martin^93^	https://combine-lab.github.io/salmon/
Samtools v1.17	Li et al.^94^	http://www.htslib.org/
R v4.0.4 & R studio	R Project	https://www.r-project.org/
Sequencing analysis pipelines	This study	https://github.com/martienssenlab/R-loop-manuscript

## Highlights

- Dicer binds R-loops and DNA:RNA hybrids
- Dicer’s helicase activity prevents genome instability when R-loops accumulate
- Dicer terminates aberrant RNA polymerase II at promoter-proximal pause sites
- Dicer cooperates with RNase H to generate small RNA at promoters
