# Supplementary material for: Transcription-replication conflict resolution by nuclear RNA interference
Source: Mol Cell. Author manuscript; Available in PMC 2025 Nov 18. (PMC12624956; doi:10.1016/j.molcel.2025.10.003)
Supplement: MMC1 [file NIHMS2119089-supplement-MMC1.pdf]

Molecular Cell, Volume *85*

Supplemental information

Transcription-replication conflict resolution

by nuclear RNA interference

Teri Cheng, Benjamin Roche, Farida Abderahmane, Leila Touat-Todeschini, Karine Fréon, Asad A. Lakhani, Sonali Bhattacharjee, Liam G. Spielmann, Emerson Jenen, Christine Choi, Jie Ren, André Verdel, Sarah A.E. Lambert, and Robert A. Martienssen

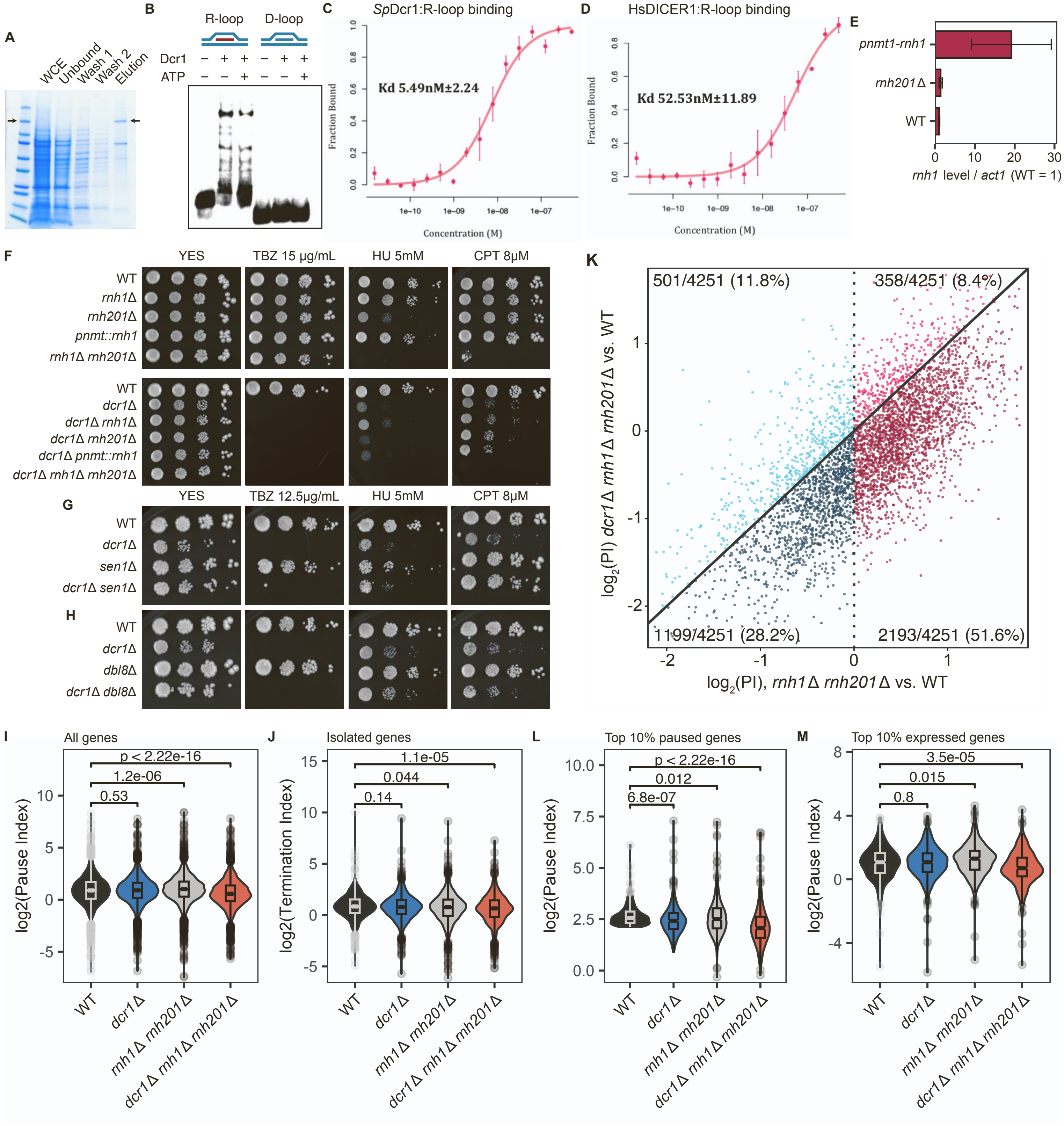
A
WCE
Unbound
Wash 1
Wash 2
Elution
B
R-loop
D-loop
Dcr1
ATP
C
SpDcr1:R-loop binding
Kd 5.49nM±2.24
Fraction Bound
Concentration (M)
D
HsDICER1:R-loop binding
Kd 52.53nM±11.89
Fraction Bound
Concentration (M)
E
pnmt1-rnh1
rnh201Δ
WT
rnh1 level / act1 (WT = 1)
F
YES
TBZ 15 μg/mL
HU 5mM
CPT 8μM
WT
rnh1Δ
rnh201Δ
pnmt::rnh1
rnh1Δ rnh201Δ
WT
dcr1Δ
dcr1Δ rnh1Δ
dcr1Δ rnh201Δ
dcr1Δ pnmt::rnh1
dcr1Δ rnh1Δ rnh201Δ
G
YES
TBZ 12.5μg/mL
HU 5mM
CPT 8μM
WT
dcr1Δ
sen1Δ
dcr1Δ sen1Δ
H
WT
dcr1Δ
dbl8Δ
dcr1Δ dbl8Δ
K
501/4251 (11.8%)
358/4251 (8.4%)
1199/4251 (28.2%)
2193/4251 (51.6%)
log2(PI) dcr1Δ rnh1Δ rnh201Δ vs. WT
log2(PI), rnh1Δ rnh201Δ vs. WT
I
All genes
p < 2.22e-16
1.2e-06
0.53
log2(Pause Index)
WT
dcr1Δ
rnh1Δ rnh201Δ
dcr1Δ rnh1Δ rnh201Δ
J
Isolated genes
1.1e-05
0.044
0.14
log2(Termination Index)
WT
dcr1Δ
rnh1Δ rnh201Δ
dcr1Δ rnh1Δ rnh201Δ
L
Top 10% paused genes
p < 2.22e-16
0.012
6.8e-07
log2(Pause Index)
WT
dcr1Δ
rnh1Δ rnh201Δ
dcr1Δ rnh1Δ rnh201Δ
M
Top 10% expressed genes
3.5e-05
0.015
0.8
log2(Pause Index)
WT
dcr1Δ
rnh1Δ rnh201Δ
dcr1Δ rnh1Δ rnh201Δ

Figure S1. Additional information on Dicer-specific interaction with R-loops. Related to Fig. 1.
(A) Coomassie gel staining showing the purification of Dcr1. The arrow on the left indicates the marker at 150 kDa, and the right indicates the Dcr1 purified at the correct size (~158 kDa).
(B) EMSA binding assay of Dcr1 with R-loops or D-loops *in vitro*, with or without ATP.
(C,D) Microscale thermophoresis (MST) binding assay of (b) *S. pombe* Dcr1 and (c) human Dicer with R-loops.
(E) RT-qPCR quantification of the expression of *rnh1* in WT, *rnh201*Δ, and the RNase H1-overexpressing strain. Expression levels were normalized to *act1*, WT = 1. N = 2. Error bars represent SEM.
(F) Spot assay of WT and *dcr1*Δ, in combinations with *rnh1*Δ, *rnh201*Δ, *rnh1*Δ *rnh201*Δ, or an RNase H1-overexpressing strain *pnmt1::rnh1*. Cells were spotted with 10-fold dilution on YES plates without supplement or with varying doses of TBZ, HU, and CPT.
(G,H) Spot assay of WT, *dcr1*Δ with *sen1*Δ (g) or *dbl8*Δ (h) with 10-fold dilution on YES plates without supplement or with varying doses of TBZ, HU and CPT.
(I) Violin plot of pausing indices (PIs) computed for all annotated genes from PRO-seq data. P-values represent results of one-way ANOVA.
(J) Violin plot of termination indices (TIs) computed from PRO-seq data. P-values represent results of one-way ANOVA. Isolated genes were used to limit confoundment from read counts from neighboring genes.
(K) Scatter plot of change in PI between the triple mutant (*dcr1*Δ *rnh1*Δ *rnh201*Δ) and WT against that between the double mutant (*rnh1*Δ *rnh201*Δ) and WT. Each dot represents an individual Pol II-transcribed gene. Dots to the right of dotted line indicate genes that increased in pausing when *rnh1* and *rnh201* were deleted, which represented ~60% of genes. Dots below the solid black line (x = y) represent genes that pausing was further reduced when *dcr1* was deleted in the RNase H-deficient double mutant, which were ~80% of genes, suggesting that Dcr1 had a global effect in mediating pausing.
(L,M) Violin plots of PIs computed for all (j) top 10% paused genes and (k) top 10% expressed genes from PRO-seq data. P-values represent results of one-way ANOVA. Top 10% paused genes were determined from WT PRO-seq data, and top 10% expressed genes were determined from WT RNA-seq data, both generated in this study.

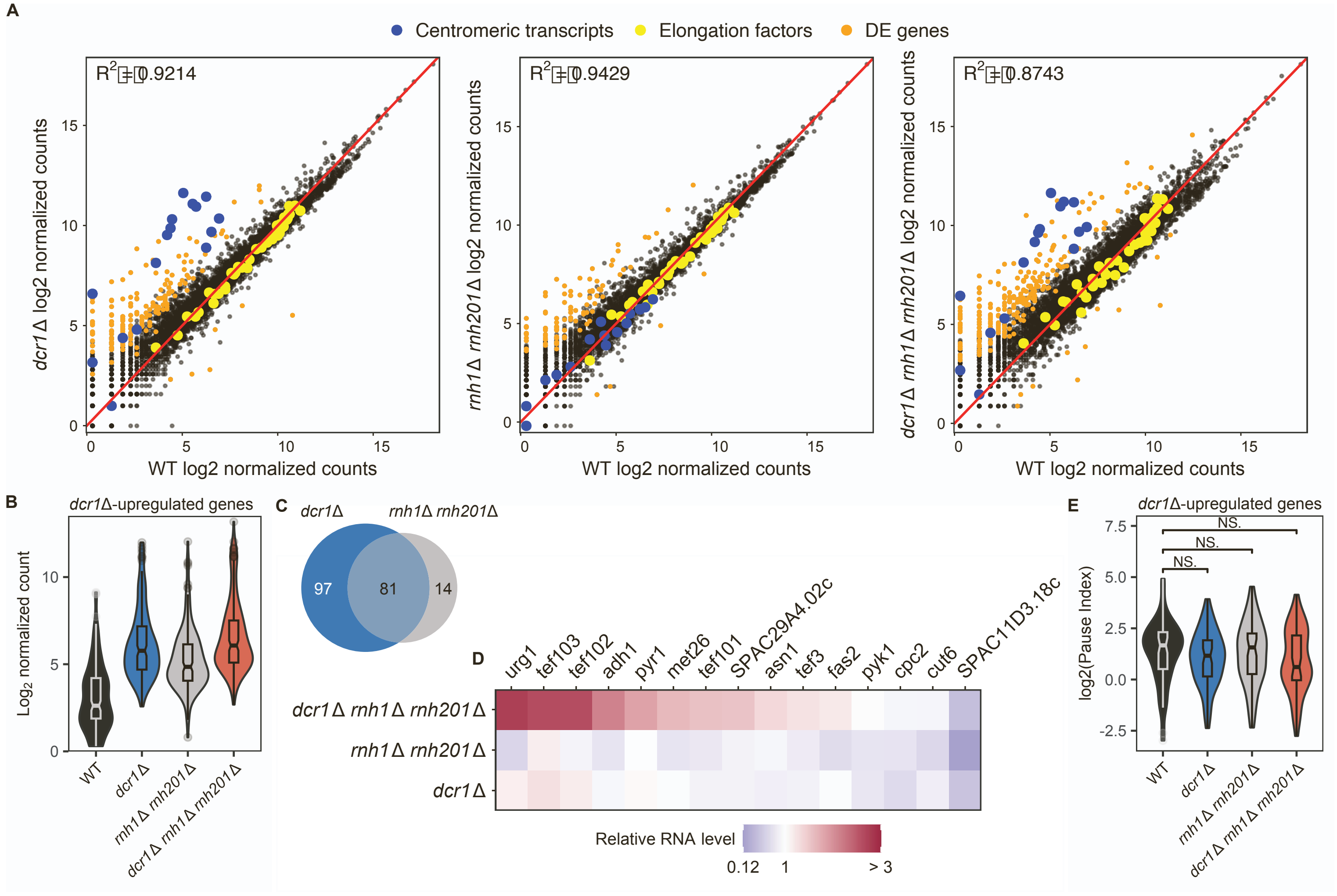

Figure S2. RNA-seq analysis of gene expression level changes in the mutantts. Related to Fig. 1.

(**A**) Correlation of normalized RNA-seq read counts for each gene (N = 6,387) between WT and dcr1Δ (left), rnh1Δ rnh201Δ (middle), and dcr1Δ rnh1Δ rnh201Δ (right). Blue dots indicate centromeric transcripts, defined as annotated genes within the centromere on PomBase. Yellow dots highlight transcription elongation factors (GO:0008023) and termination factors (GO:0006369). Orange dots are significantly differentially expressed genes, defined as Log_2 fold-change > 2, and with adjusted p-values < 0.05.

(**B**) Violin plot of log_2 normalized expression of dcr1Δ upregulated genes in various mutants. dcr1Δ upregulated genes were defined as in (a).

(**C**) Venn diagram of genes upregulated in dcr1Δ and in rnh1Δ rnh201Δ showing a largely overlapping list of genes mis-regulated in both mutants.

(**D**) Heatmap showing the relative RNA count, normalized to WT, of 15 genes upregulated specifically in the triple mutant dcr1Δ rnh1Δ rnh201Δ.

(**E**) Violin plot of pausing indices (PIs) of *dcr1*Δ-upregulated genes. P-values represent results of one-way ANOVA. NS. Not significant.

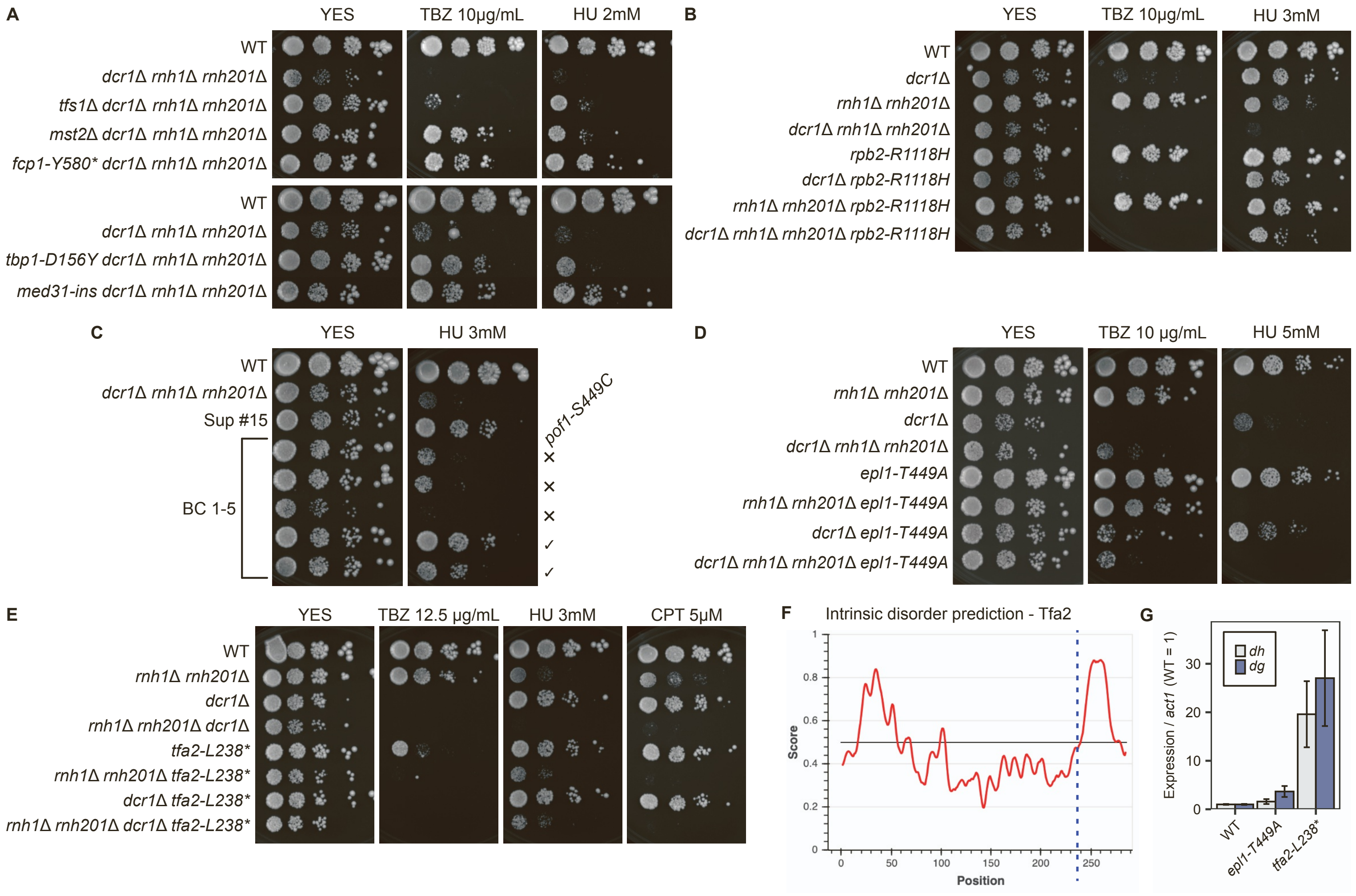

Figure S3. Genetic suppressors of *dcr1Δ*. Related to Fig. 2.

(**A**) Spot assay of the triple mutant *dcr1Δ rnh1Δ rnh201Δ* in combination with various alleles of general transcription factors. Cells were plated with 10-fold dilution on YES plates without supplement or with TBZ or HU to test for drug sensitivity.

(**B**) Spot assay to verify *rpb2-R1118H* as a suppressor. Cells were plated with 10-fold dilution on YES plates without supplement or with TBZ or HU to test for drug sensitivity.

(**C**) Spot assay to verify the *pof1-S449C* allele as the causative SNP in suppressor #15. Cells were plated with 10-fold dilution on YES plates without supplement or with HU to test for drug sensitivity. SNPs in backcrosses (BC) were tested using dCAPs (see methods).

(**D**) Spot assay to verify *epl1-T449A* as a suppressor. Cells were plated with 10-fold dilution on YES plates without supplement or with TBZ or HU to test for drug sensitivity.

(**E**) Spot assay to verify *tfa2-L238** as a suppressor. Cells were plated with 10-fold dilution on YES plates without supplement or with TBZ, HU, or CPT to test for drug sensitivity.

(**F**) IUPred3 prediction of Tfa2 (UniProt: P79011) showing the disorder tendency. Vertical blue dotted line represents the approximate location of residue L238, that which was turned into a premature stop codon in LS22.

(**G**) RT-qPCR quantification of the expression of *dg/dh* centromeric transcripts in *epl1-T449A* and *tfa2-L238**. Relative expression was normalized to *Act1*. N = 2 independent biological replicates. Error bars represent SEM.

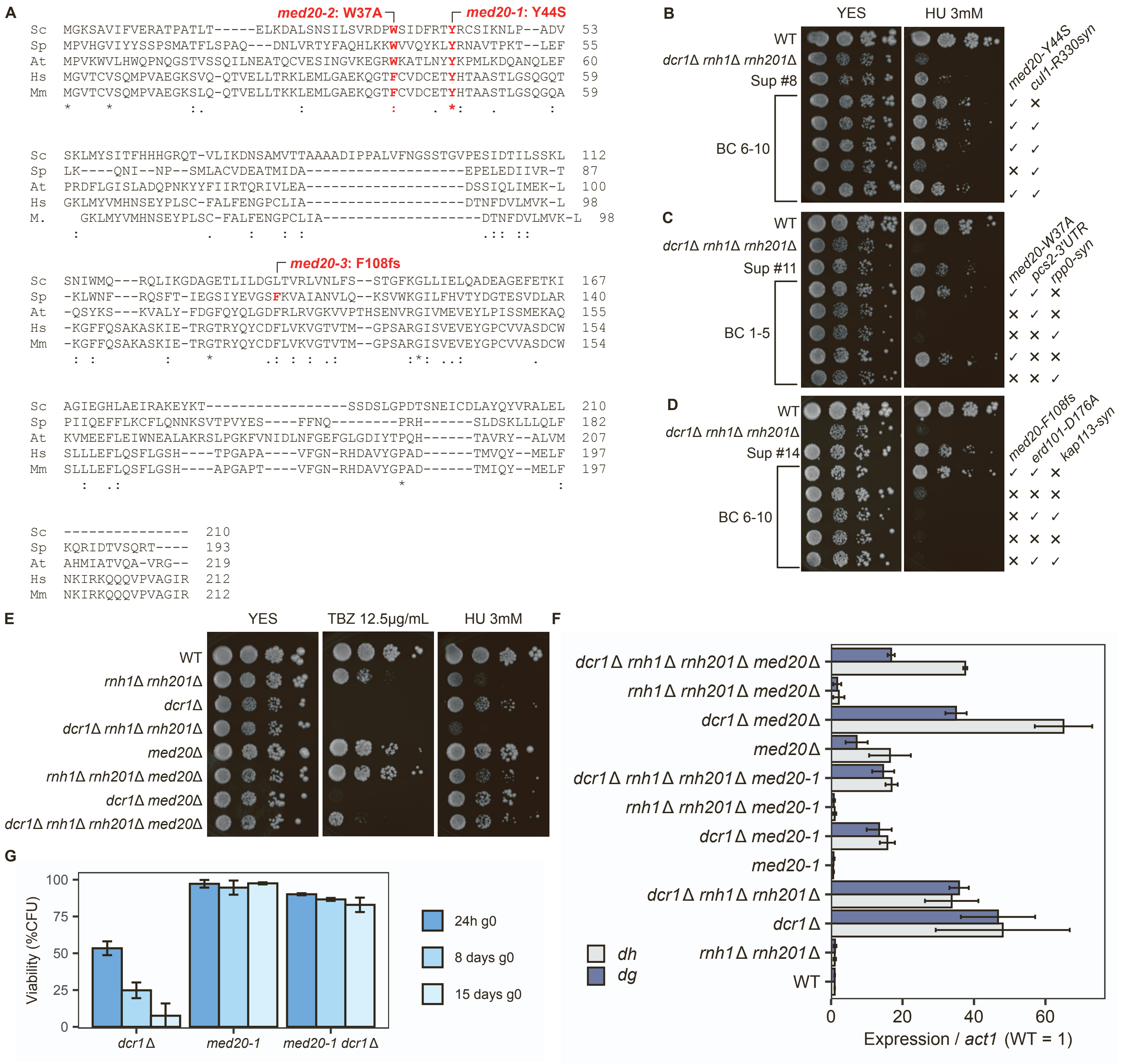

Figure S4. Additional characterization of suppression by *med20* alleles. Related to Fig. 2.
(**A**) Sequence alignment of *med20* from *Saccharomyces cerevisiae* (Sc), *S. pombe* (Sp), *Arabidopsis thaliana* (At), *Homo sapiens* (Hs), and *Mus musculus* (Mm). Alignment was done using Clustal Omega with default setting. SNPs for *med20-1, med20-2,* and *med20-3* were highlighted in red.
(**B-D**) Backcross verification of (b) *med20-1,* (c) *med20-2*, and (d) *med20-3* alleles respectively. Cells were plated with 10-fold dilution on YES plates without supplement or with HU to test for drug sensitivity. SNPs in backcrosses (BC) were tested using dCAPs (see methods).
(**E**) Spot assay to test the suppression activity of *med20*Δ. Cells were plated with 10-fold dilution on YES plates without supplement or with TBZ or HU to test for drug sensitivity.
(**F**) RT-qPCR quantification of the expression of *dg/dh* centromeric transcripts in various mutants. Fold change was calculated relative to *Act1* and WT. N = 2 - 3 independent biological replicates. Error bars represent SEM. Note only *med20*Δ but not *med20-1* has silencing defect, and that *med20-1* could mildly suppress silencing defect in *dcr1*Δ.
(**G**) Viability assay of G_0 entry and quiescence maintenance in prototroph *dcr1*Δ, *med20-1*, and *med20-1 dcr1*Δ. *dcr1*Δ has severe G_0 entry and maintenance defect ref. [11], and *med20-1* was able to almost fully suppress this phenotype of *dcr1*Δ. Data for *dcr1*Δ was adapted from ref. [11].

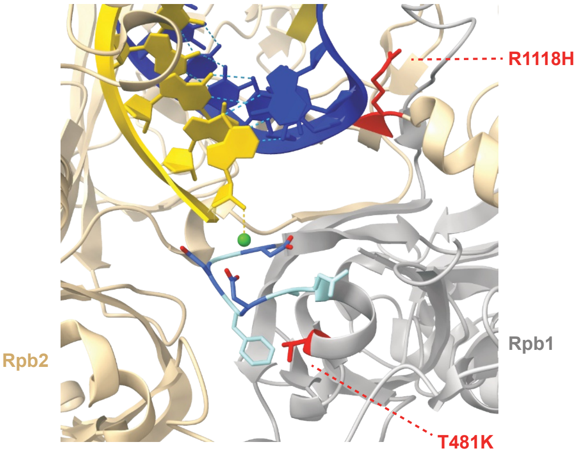

Figure S5. rpb1-T481K and rpb2-R1118H disrupt the catalytic core of Pol II. Related to Fig. 2.
Catalytic center of Pol II highlighting the two residues in red, Rpb1-T481K and Rpb2-R1118H, whose mutations suppress dcr1Δ. Rpb1 and Rpb2 are colored in gray and beige respectively. Note that Rpb1-T481 aligns with the catalytic triad (NADFDGD, in light blue) coordinating a Mg^{2+} ion (green) close to RNA (yellow), and that Rpb2-R1118 is in proximity to the template strand (TS) DNA (blue). Image generated from PDB:5vvs using ChimeraX.

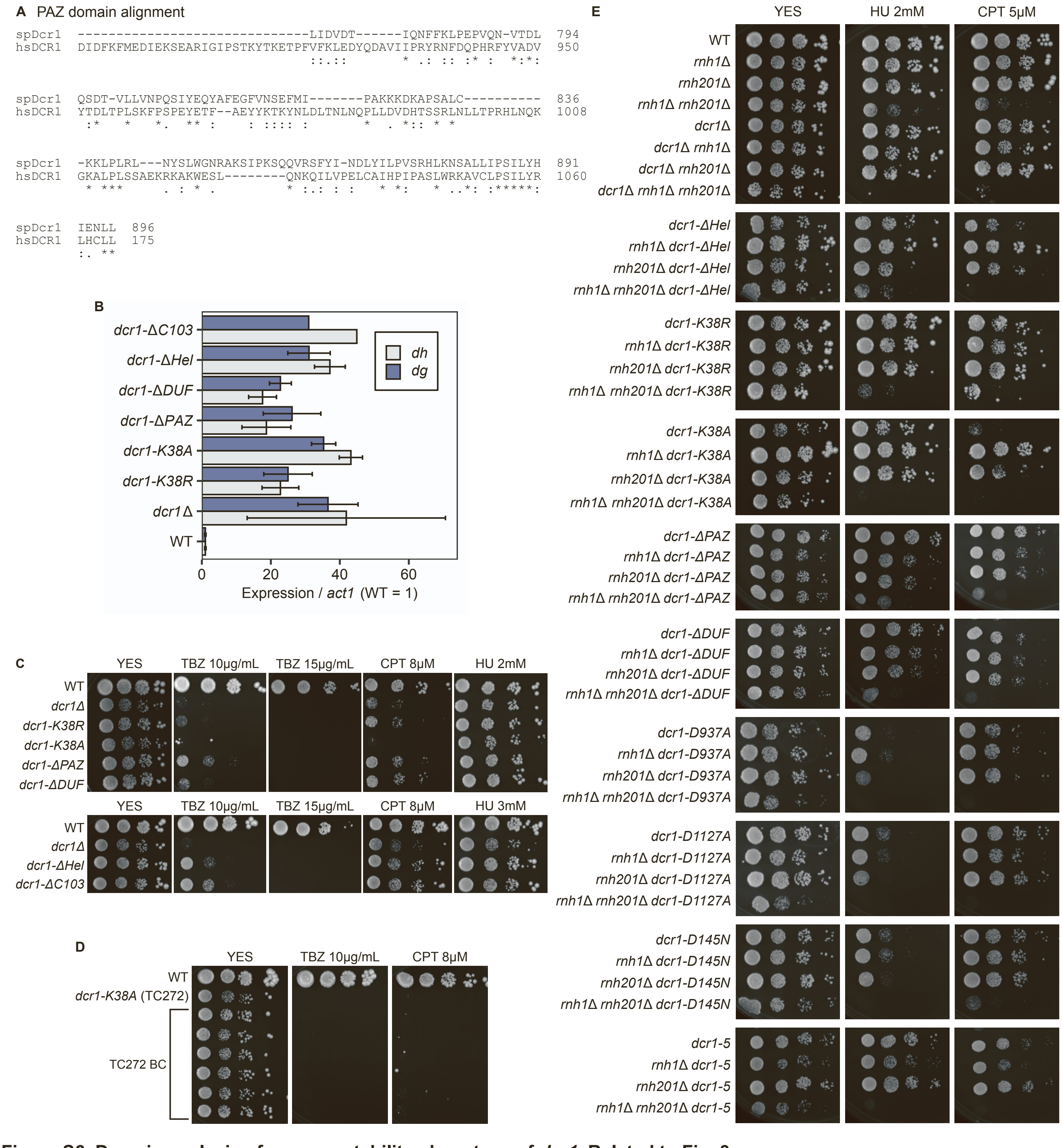

Figure S6. Domain analysis of genome stability phenotype of *dcr1*. Related to Fig. 3.

(**A**) Sequence alignment of the PAZ domain of human Dicer with parts of *S. pombe* Dcr1, showing the region deleted for *dcr1-ΔPAZ* in this study.

(**B**) RT-qPCR quantification of the expression of *dg/dh* centromeric transcripts in various *dcr1* alleles. Fold change was calculated relative to *Act1* and WT. N = 3 independent biological replicates. Error bars represent SEM. All *dcr1* alleles lost silencing, indicating the entire *dcr1* was important for centromeric function.

(**C**) Spot assay of various *dcr1* alleles. Cells were plated with 10-fold dilution on YES plates without supplement or with TBZ or CPT to test for drug sensitivity. All *dcr1* alleles were sensitive to TBZ, although to slightly varying extent. Note only *dcr1-K38A* was acutely sensitive to CPT, worse than *dcr1*Δ.

(**D**) Backcross analysis of TC272, a strain carrying the *dcr1-K38A* allele alongside a cassette conferring hygromycin resistance. Hygromycin-resistant colonies were selected from backcross and the SNP for *dcr1-K38A* was confirmed using dCAPs.

(**E**) Spot assays of various *dcr1* alleles crossed to *rnh1*Δ and *rnh201*Δ. Cells were plated with 10-fold dilutions on YES plates without supplement or with HU or CPT to test for drug sensitivity. All *dcr1* alleles negatively interacted with *rnh1*Δ *rnh201*Δ, including the *dcr1-5* allele, suggesting the function from an intact Dcr1 was required for genome maintenance.

Table S3. Oligonucleotide sequences for biochemical assays. Related to Fig. 1 and S1.

Name	Sequence (5'-3')	Use
DNA1	GGGTGAACCTGCAGGTGGGCGGCTGCTCATCGTAGGTTAGTTGGTAGAATTCGGCAGCGTC	Anneal with DNA2 for R-loops
DNA2	GACGCTGCCGAATTCTACCAGTGCCTTGCTAGGACATCTTTGCCCACCTGCAGGTTCACCC	DNA:RNA and R-loops
RNA	AAAGAUGUCCUAGCAAGGCAC	Anneal with DNA2 for DNA:RNA and R-loops

Table S4. EMS suppressor screen for HU-resistance. Related to Fig. 2. Causative SNPs were verified with either co-segregation analysis through backcrosses, or by reintroduction into the triple mutant. When the causative SNPs were not verified, the most likely SNP(s) among the candidates were highlighted. N.D.: Not determined.

Suppressor	Sterile?	Causative SNP	Candidate SNPs
LS15	No	**Epl1-T449A**	SPNCRNA.857 (chr1:2872396 A->G); Map4-S326syn; Prl10 (chr3:1839843 C->G); an intergenic SNP (chr2:3020359 C->G)
LS16	Yes	**Rpb2-R1118H**	Esc1-S338C; Gas1-S516syn; Sea3-H321R; Syt22-R526G; Sld5-G192A; Fes1-D232V; Dbp8-R345Q; Snp22-Q269P
LS17	N.D.	-	Many (37 SNPs)
LS18	Yes	-	**Utp5-E601D; Ubr1-P1524S**
LS19	N.D.	-	3 SNPs on **Edc1** leading to truncation; Synonymous SNPs on Mde5, SPNCRNA.253, Wtf1, and Rga3
LS20	No	**Med20-Y44S**	Cul1-R330syn; a tRNA gene (chr2:820558 C->T)
LS21	Yes	**Rpb1-T481K**	Tlg2 (5'UTR); Agl1-G491A; Eno101 (5'UTR); Ptr3-P289A; Osh6 (intron); an intergenic SNP (chr1:4269071 T->C)
LS22	N.D.	**Tfa2-L238***	Nup132 (5'UTR); SPAC17A2.11 (5'UTR); Mas5-E362V; Tan1-I63P; Tif11 (3'UTR); Hrf1 (3'UTR); Vac14-N252K; Rrg1-S119P; SPCC18.17c-L18*
LS23	No	**Med20-W37A**	Pcs2 (3'UTR); Rpp0 (synonymous); one intergenic SNP (chr2:4471290 C->G)
LS24	No	-	**Cts1-V58L**; Spp42 (synonymous); Saf1 (3'UTR)
LS25	No	-	**Rok1-A388T**; Nte1-K806Q; Rpt3-D234A; one intergenic SNP (chr1:3022972 C->T)
LS26	No	**Med20 truncation**	Erd101-D176A; Kap113 (intron); one intergenic SNP (chr1:4415439 G->T)
LS27	No	**Pof1-S449C**	-
LS28	Yes	-	Gas5-V219syn; SPBC359.01-V342G; **Med15-L63***; SPBC83.05 (3'UTR); Map2 (5'UTR)
